# A rapid passage through a two-active-X-chromosome state accompanies the switch of imprinted X-inactivation patterns in mouse trophoblast stem cells

**DOI:** 10.1186/s13072-015-0044-2

**Published:** 2015-12-01

**Authors:** Julie Prudhomme, Agnès Dubois, Pablo Navarro, Danielle Arnaud, Philip Avner, Céline Morey

**Affiliations:** Mouse Molecular Genetics Laboratory, Pasteur Institute, 25 rue du Dr Roux, 75015 Paris, France; Epigenetics of Stem Cells Laboratory, Pasteur Institute, 25 rue du Dr Roux, 75015 Paris, France; Dynamics of Epigenetic Regulation, EMBL Monterotondo, Adriano Buzzati-Traverso Campus, Via Ramarini 32, 00015 Monterotondo, Italy; CNRS, UMR7216 Epigenetics and Cell Fate, 35 rue Hélène Brion, 75013 Paris, France

**Keywords:** Imprinted X-inactivation, Trophoblast Stem cells, Epigenetic Reprogramming

## Abstract

**Background:**

In female mice, while the presence of two-active X-chromosomes characterises pluripotency, it is not tolerated in most other cellular contexts. In particular, in the trophoblastic lineage, impairment of paternal X (X^P^) inactivation results in placental defects.

**Results:**

Here, we show that Trophoblast Stem (TS) cells can undergo a complete reversal of imprinted X-inactivation without detectable change in cell-type identity. This reversal occurs through a reactivation of the X^P^ leading to TS clones showing two active Xs. Intriguingly, within such clones, all the cells rapidly and homogeneously either re-inactivate the X^P^ or inactivate, de novo, the X^M^.

**Conclusion:**

This secondary non-random inactivation suggests that the two-active-X states in TS and in pluripotent contexts are epigenetically distinct. These observations also reveal a pronounced plasticity of the TS epigenome allowing TS cells to dramatically and accurately reprogram gene expression profiles. This plasticity may serve as a back-up system when X-linked mono-allelic gene expression is perturbed.

**Electronic supplementary material:**

The online version of this article (doi:10.1186/s13072-015-0044-2) contains supplementary material, which is available to authorised users.

## Background

In male mammals, the XY genotype results in the mono-allelic expression of X-linked genes. In contrast, in females, the presence of two X-chromosomes may lead to bi-allelic X-linked expression, which is known to be detrimental to the embryo [1]. To prevent this double dose of X-linked products, female mammals inactivate one X-chromosome. In the mouse, two forms of X-inactivation exist. A completely biased inactivation of the X^P^ is first established in the 4-cell embryo [2–4]. At the morula–blastocyst transition, the X^P^ is reactivated in cells of the inner cell mass (ICM)—one of the two cell types known to be able to withstand two active Xs—while the inactivation of the X^P^ persists in the extraembryonic lineages of the trophectoderm and of the primitive endoderm and in their placental and yolk sac derivatives, respectively [3, 5–7]. As opposed to this imprinted inactivation (I-XCI), upon epiblast formation, ICM cells independently choose to inactivate either the X^P^ or the maternal X (X^M^) at random [8]. This initial choice is then clonally inherited thereby giving rise to an allelic mosaicism of X-linked gene expression within female adult tissues. This random X-inactivation (R-XCI) is extremely stable and is only reverted in germ cells, which, therefore, constitute the second cell type known to lack XCI.

Both I-XCI and R-XCI rely on the same basic mechanism: the overexpression of the *Xist* gene from the future inactive X and a *cis*-accumulation of *Xist* ncRNAs, which triggers a cascade of epigenetic changes ending up in the formation of a heterochromatic X-chromosome (for review see [9, 10]). Beyond this common core mechanism, lineage-specific differences in the establishment and stability of the inactive state have been investigated in vivo, during the blastocyst development, but also ex vivo, using cellular models of the three blastocyst lineages, namely the embryonic stem (ES) cells [11], the trophoblast stem (TS) cells [12] and the extraembryonic endoderm stem (XEN) cells [13]. Intriguingly, amongst these different cell types, there seems to be a correspondence between the cell potency, the degree of stability of the inactive state and the level of tolerance of X-linked bi-allelic expression. Pluripotent ES cells stand at the extremity of this continuum since they relatively happily maintain two active Xs. A control of X-inactivation initiation by pluripotency markers and, reciprocally, a stabilisation of the naïve pluripotent state by two active X-chromosomes have been suggested to sustain this equilibrium [14, 15].

In contrast, the multipotent trophoblast cells appear especially refractory to any global deregulation of X-chromosome expression since bi-allelic X-linked gene expression in the trophectoderm of embryos carrying mutations in paternal alleles of *Xist* results in lethality due to extraembryonic defects [16, 17]. Paradoxically, this latter lineage is particularly rich in gene escaping from I-XCI—i.e. genes expressed from both Xs—compared to other adult cell types [18, 19]. In addition to this, transient and spontaneous reactivations of certain X-linked genes occur both and ex vivo [20] and, after differentiation, the relaxation of I-XCI extends to additional genes in specific subtypes of placental cells [21–25]. Even more dramatically, complete inversion of XCI profiles has been observed in few spongiotrophoblast progenitor cells before the appearance of global placental defects in embryos carrying a paternal *Xist* mutation [26]. Since the X-chromosome is enriched in genes involved in placental functions compared to most autosomes [27], these observations suggest that the trophectoderm is the site of an opposition between the requirement for X-chromosome plasticity of expression necessary to commit into multiple trophoblastic fates and the need to maintain specific X-linked genes under a tight dosage control in specific subtypes of placental cells to ensure that the placenta functions properly.

In order to understand how the plasticity of X-chromosome expression is regulated in the trophectoderm lineage, we used TS cells carrying a mutation in the maternal X-linked gene *Hprt1.* In this context, the expression of *Hprt1* serves as an index of X-chromosome activity and cells that re-express the unmutated, paternal, *Hprt1* copy can be selected for with aminopterin (HAT medium). Using this system, we isolated, among others, HAT-resistant clones showing a reversal of I-XCI profiles characterised by an inactive X^M^ and a reactivated X^P^. This reversal is mediated by a passage through a two-active-X state and followed by a de novo inactivation of the X^M^ involving an accumulation of *Xist* RNAs on the chromosome and a recruitment of H3K27me3 silent histone mark at most—but not all—X-linked genes. Importantly, within clonal cell populations showing two-active X-chromosomes, all cells homogeneously choose to inactivate the same chromosome: the X^P^ in most clones, or the X^M^ in rare instances. No mosaic clones constituted of a mixture of cells with an inactive X^M^ and of cells with an inactive X^P^ have been observed. This suggests that the choice process used, in TS cells, after the initial reactivation of the X^P^ is different from a random X-chromosome choice as it is described to occur upon differentiation of epiblast cells or upon differentiation of ES cells. This furthermore indicates that the two-active-X state in TS cells is epigenetically different from the two-active-X state in pluripotent cells.

## Results

### Cells expressing the *Hprt1* gene from the paternal X-chromosome pre-exist in undifferentiated populations of female TS cells

In order to evaluate the stability of I-XCI in undifferentiated TS cells, we used a female TS cell line (F3) carrying a mutation at the X-linked *Hprt* locus (*Hprt*^*bm1*^) associated with a retroviral insertion in the maternal *Hprt1* gene, which leads to a stable loss-of-function of the maternal allele [28]. In such a genetic configuration, the expression of the *Hprt1* locus can be theoretically used as an index of X^P^ and X^M^ activities by growing cells on two different selective media. Growth on medium complemented with HAT (Hypoxanthine Aminopterin Thymidine) selects for cells expressing a functional, paternal, *Hprt1*^*WT*^ allele, while only cells unable to use the regular HPRT1 pathway, which have, therefore, supposedly, inactivated the paternal *Hprt1*^*WT*^ copy, will survive on 6-TG (6-ThioGuanine) medium. In addition, the F3 cell line carries an X^M^ of 129Sv.Pgk1a origin and an X^P^ of 129Sv origin allowing the identification of allele origin through the extensive polymorphisms located in the large Pgk1a-derived region surrounding the *Xist* gene.

**Fig. 1:**
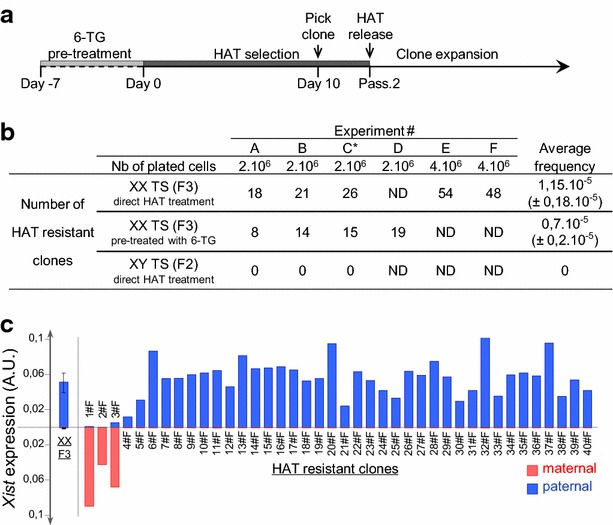
HAT-resistant clones of F3 female TS cells show various patterns of *Xist* expression including an inverted *Xist* expression profile. **a** Protocol of isolation of HAT-resistant TS clones. Female F3 TS cells were either directly treated with HAT or pre-treated with 6-TG before HAT selection. HAT-resistant clones were picked at day 10 of selection and maintained under HAT selection for 2 additional passages. Analysis of *Xist* expression was performed after 8 passages. **b** Table showing the frequencies of HAT-resistant clones obtained in the time course of 6 independent experiments (#*A*, #*B*, #*C*, #*D*, #*E* and #*F*). The frequency of HAT-resistant clones after pre-treatment with 6-TG is significantly different from the frequency of HAT-resistant clones in the absence of 6-TG pre-treatment (Mann–Whitney test, *p* value <0.05). The asterisk indicates that in experiment #*C,* the HAT selection has been maintained for a longer period of time, see Additional file 5A. The protocol described in *panel* **a** has been applied in the other experiments (i.e. #*A*, #*B*, #*D*, #*E,* and #*F*). In this study, each individual clone analysed is identified by a capital letter which refers to the experiment from which the clone has been isolated and by a number which identifies each clone of a given experiment. Clones that have been characterised further in this study all come from HAT selection without 6-TG pre-treatment. **c** Cumulative histograms showing the *Xist* expression levels measured by allelic RT-qPCR after standardisation by the *Rplp0* reporter gene [20] in the 40 clones obtained in experiment #F (passage 8 after clone picking). Paternal and maternal expressions are depicted in *blue* and *red* above and below the *x*-axis, respectively. For F3 parental female cells, mean and standard deviation have been calculated on the basis of three independent cell cultures. *A.U.* Arbitrary Units

We first determined the frequency of spontaneous reversal of the [Hprt-] phenotype (previously estimated to be <10^−8^ in primary fibroblasts [28]) by growing *Hprt*^*bm1*^ male TS cells (F2 cell line) on HAT medium (Fig. 1a, b). No HAT-resistant clones were observed in the time course of three independent experiments (experiments #A, #B and #C), indicating that the rate of [Hprt−] spontaneous reversal is very low in TS cells and will not significantly influence our results (Fig. 1b). Similarly, the vast majority of F3 female TS cells died upon HAT selection confirming that the paternal *Hprt1*^*WT*^ allele is silenced by I-XCI in most cells. A number of HAT-resistant F3 clones were, however, observed with a consistent frequency of ~10^−5^ over five independent experiments (experiments #A, #B, #C, #E and #F; Fig. 1b). This suggests that some female F3 TS cells have reactivated the paternal *Hprt*^*WT*^ allele.

In order to rule out the possibilities that these HAT-resistant clones were all descendants of rare events that occurred at the time of F3 cell line derivation or that the HAT treatment, by itself, triggers the paternal *Hprt1*^*WT*^ reactivation, we first sub-cloned the F3 cell line and tested four independent sub-clones for their ability to generate HAT-resistant cells. Significant frequencies of [Hprt−] reversal were obtained with all four sub-clones indicating that each F3 TS cell has a similar probability and ability to reactivate the paternal *Hprt1*^*WT*^ allele (data not shown). Secondly, we grew F3 TS cells on 6-TG prior to HAT selection in order to eliminate potential pre-existing [Hprt+] cells from the population before HAT selection. Under these conditions, we still recovered HAT-resistant clones with a significant frequency (~0.7 × 10^−5^) compared to spontaneous [Hprt−] reversal in F2 male cells again confirming that F3 TS cells are homogeneously able to induce *Hprt1*^*WT*^ reactivation (experiments #A, #B, #C and #D; Fig. 1b). This frequency, however, was significantly and reproducibly lower than the frequency obtained upon direct HAT selection suggesting that TS cells displaying an active *Hprt1*^*WT*^ paternal allele are naturally present amongst the F3 cell population.

Importantly, HAT-resistant clones appeared morphologically indistinguishable from parental F3 cells (see Additional file 1A). In addition, when we analysed the expression of classical TS cell markers and markers specific for differentiated TS states in HAT-resistant cells, most clones showed expression profiles similar to undifferentiated F3 cells or to the other TS cell lines we tested, indicating that these HAT-resistant cells displayed the characteristics of bona fide TS cells (see Additional file 1B). We concluded from these observations that female TS cells are prone to spontaneously reactivate the *Hprt1*^*WT*^ locus on the paternal X-chromosome and recurrently do so at a low but significant frequency during cell growth.

### Reactivation of the paternal *Hprt1* locus is accompanied by a switch of X-inactivation profiles in a subset of HAT-resistant TS cells

In order to determine the extent of X^P^ reactivation amongst various HAT-resistant clones and, in particular, establish the frequency of clones that may have reactivated the entire X^P^, we analysed the expression of *Xist* using allelic RT-qPCR in all the clones recovered during the time course of a single HAT selection (experiment #F). Strikingly, we observed two distinct *Xist* expression profiles: 37 out of 40 clones displayed paternal *Xist* expression levels similar to parental F3 cells, while 3 clones showed a maternally restricted expression of *Xist* (Fig. 1c). When we performed *Xist* RNA-FISH on representative clones of each category, we observed a single *Xist* domain in most nuclei of both categories (see Additional file 2A). This suggests that the clones expressing *Xist* from the X^P^ locally reactivated the paternal *Hprt1*^*WT*^ gene. This is confirmed by the transcription of the *Hprt1*^*WT*^ alleles from the *Xist*-coated X-chromosome in a significant percentage of cells of these clones (as determined by RNA-FISH) and by a preferential expression of maternal alleles of other X-linked genes as assessed both by allelic RT-qPCR and by RNA-FISH (see Additional file 2B, C). We noticed variability in the percentage of cells transcribing the *Hprt1*^*WT*^ allele between clones (see Additional file 2C). Since the analysis has been performed several passages after HAT release, this suggests that the *Hprt1*^*WT*^ allele has been silenced de novo in a percentage of cells that varies from clone to clone. Similar transient and local reactivations of *Hprt1* and of several X-linked loci on the inactive X^P^ have been previously reported to occur spontaneously and at low frequency in another TS cell line and in the cells of the Trophectoderm in vivo [20].

**Fig. 2:**
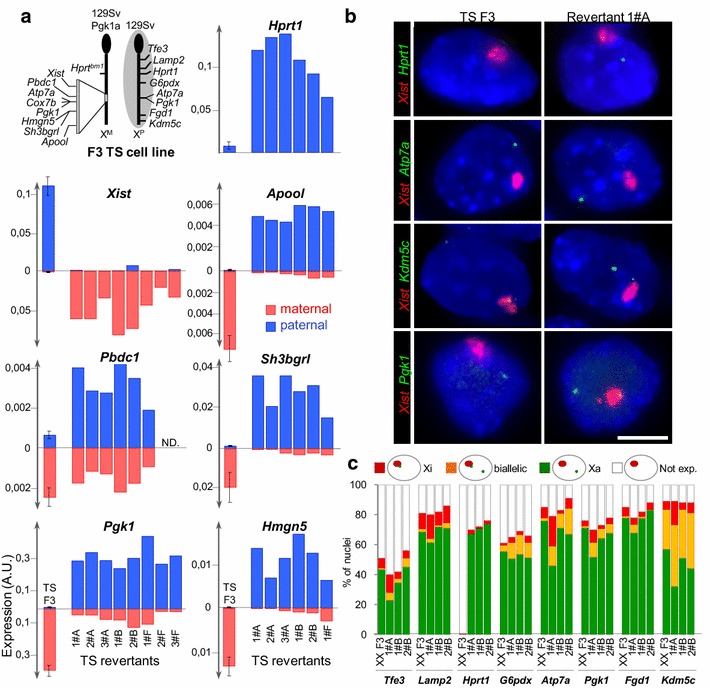
Allelic inversion of *Xist* expression is associated with a reversal of X-inactivation profiles. **a** Cumulative histograms showing the expression levels of several X-linked genes located within the 129/Pgk1a polymorphic region measured by allelic RT-qPCR in the 8 *Xist* revertant clones from independent HAT selection experiments (analysis performed at passage 8 or later after clone picking). Standardisation by *Rplp0* has been applied. Paternal and maternal expressions are depicted in blue and red above and below the *x*-axis, respectively. The diagram shows the genomic positions of the genes analysed by RT-qPCR (on the 129/Pgk1a chromosome) and by RNA-FISH (on the 129 chromosome) in *panel* **a** and **b**. For F3 parental female cells, mean and standard deviation have been calculated on the basis of three independent cell cultures. *A.U.* Arbitrary Units. **b** Representative images of double RNA-FISH for *Xist* and for the indicated X-linked gene in F3 parental cells and in cells of clone 1#A. The inactive X-chromosome is identified through *Xist* RNA accumulation (*red*). Primary transcription at the indicated X-linked gene is co-detected in *green*. <1 % of F3 parental TS cells show an *Hprt1* signal in agreement with the inactivation of the wild-type, paternal, *Hprt1* allele. In contrast, an *Hprt1* signal away from the *Xist* domain is detected in the majority of nuclei of clone 1#A indicating a reactivation of the X^P^ carrying the wild-type *Hprt1* gene. *Scale bar* = 5 μm. **c** Cumulative histograms of the percentages of nuclei with the depicted expression pattern in F3 TS cells and in nuclei of 3 independent *Xist* revertant clones (analysis performed at passage 8 or later after clone picking). See Additional file 10 for probe position. No significant difference is observed between any of the clones and F3 parental cells for all X-linked genes except for *Hprt1* (*χ* ^2^ test *p* value < 0.05). *n* > 150

We, therefore, focussed our study on the minority of clones in which the reactivation of the paternal *Hprt1*^*WT*^ allele is accompanied by a loss of paternal *Xist* expression and by an induction of maternal *Xist* RNA accumulation, which suggests that these TS cells have undergone a complete reversal of the *Xist* expression profile. To verify this hypothesis, we first analysed, by allelic RT-qPCR, the expression of eight X-linked genes located within the 129 Sv/Pgk1a polymorphic region and of *Hprt1*, in eight *Xist* revertant clones collected from independent HAT selections (Fig. 2a and see Additional file 2D; each individual clone is identified by a capital letter which refers to the experiment from which the clone has been isolated and by a number which identifies each clones within a given experiment). Most X-linked genes—except for *Pbdc1* (also called *2610029G23Rik*), which is known to escape from X-inactivation in most tissues [29]—appeared to be preferentially expressed from the X^P^. We noted however that some X-linked genes including *Pgk1*, *Cox7b* and *Atp7a* showed variable levels of residual expression from the X^M^ in these revertants. This suggests that a subset of cells within these clones still express the maternal alleles of these genes at a low level.

In parallel with these quantifications, we performed *Xist* RNA-FISH in combination with *Hprt1* (which identifies transcription from the X^P^) and 7 other X-linked genes (including loci located outside of the 129 Sv/Pgk1a polymorphic region) (Fig. 2b, c). In the three *Xist* revertant clones that were analysed, we observed a preferential mono-allelic expression from the active X for *Hprt1* and the other X-linked genes that were tested. This confirms that the X^P^ has been reactivated, while the X^M^ has been inactivated de novo in these clones. As expected, *Kdm5c,* an X-inactivation escaper [29], was expressed from both Xs.

Thus, *Xist* inverted expression profile is associated with a chromosome-wide allelic switch of X-inactivation patterns in the majority of cells of a subset of HAT-resistant TS cell clones.

### De novo X-inactivation of the maternal X-chromosome is associated with an enrichment of H3K27me3 at most, but not all, X-linked genes

In order to test whether this de novo silencing of the X^M^ in I-XCI revertant clones is accompanied by the chromatin changes associated with X-inactivation, we analysed the distribution of H3K27me3, a silent histone mark known to be recruited at the inactive X [30–32], using immunofluorescence against this histone mark combined with RNA-FISH for *Xist*. As in F3 parental TS cells, in two different revertant clones, the *Xist* domain appeared consistently associated with an accumulation of H3K27me3 (see Additional file 3A, B).

**Fig. 3:**
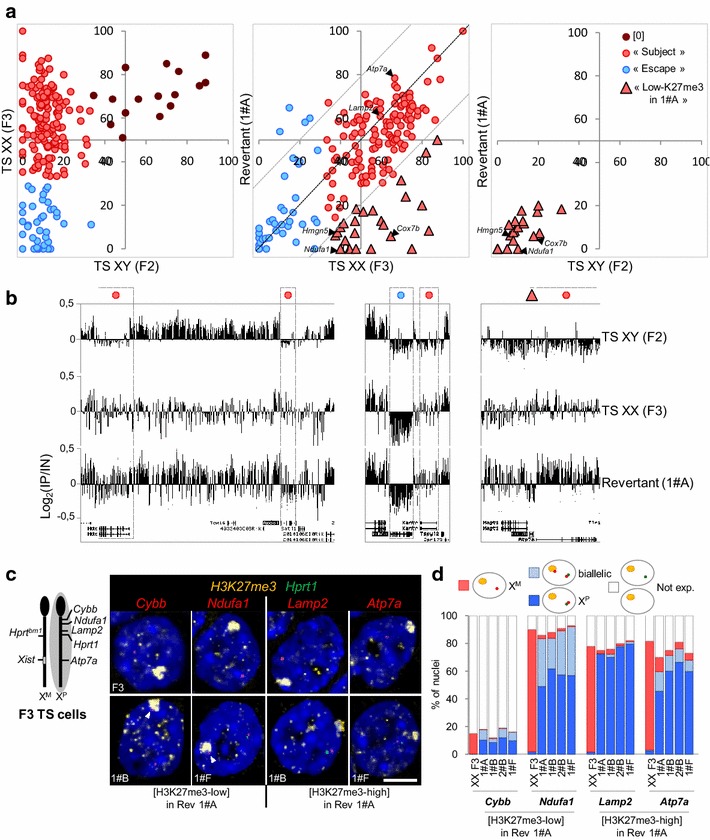
*Xist* RNA domain on the maternal inactive X in I-XCI revertant clones is associated with and accumulation of H3K27me3 at the majority of expressed X-linked genes. **a** *Left*, scatterplot of H3K27me3 percentages along the body of expressed X-linked genes in female F3 (*y*-axis) relative to male F2 (*x*-axis) TS cells. Each dot represents a single gene and its respective percentage of H3K27me3 in male and female TS cells. *k*-means clustering was applied, which led to the identification of three classes of genes. Genes subject to I-XCI (*red dots*) are concentrated in the upper left quadrant consistently, with them being depleted in H3K27me3 in male cells and enriched in H3K27me3 in female cells as previously established [20]. Genes escaping from I-XCI in TS cells (*blue dots*) are significantly depleted in H3K27me3 in both male and female cells. In agreement with H3K27me3 marking preferentially the silent state, very few expressed genes are enriched in H3K27me3 in both male and female cells ([0] genes; indicated by maroon dots). These [0] genes may have resulted from the fact that gene expression data [29] and our ChIP-chip experiment data were obtained from different TS cell lines. Middle, scatterplot of H3K27me3 percentages along the body of expressed X-linked genes in the I-XCI revertant clone 1#A (*y*-axis) relative to female F3 (*x*-axis) TS cells. Genes marked with red triangles “H3K27me3-low in 1#A” represent genes that have not regain H3K27me3 on the inactive X after the reversal of I-XCI patterns. *Right*, scatterplot of H3K27me3 percentages along the body of expressed X-linked genes in the I-XCI revertant clone 1#A (*y*-*axis*) relative to male F2 (*x*-*axis*) TS cells. “H3K27me3-low in 1#A” genes show a percentage of H3K27me3 that is not significantly different from the low levels of H3K27me3 on active allele in male TS cells by Kolmogorov–Smirnov test. The H3K27me3 profiles of *Ndufa1*, *Lamp2* and *Atp7a* analysed in *panel* **c** and **d** and of *Cox7b* and *Hmgn5* analysed in Additional file 3C are shown. **b** Representative examples of H3K27me3 distribution along expressed genes in male, female and I-XCI clone 1#A TS cells. University of California Santa Clara (UCSC) Genome Browser screenshots (mm8 build) are shown. *IP* immunoprecipitated DNA; *IN* input DNA. **c** Representative images of immuno-RNA-FISH for H3K27me3 (*yellow*) and *Hprt1* (*green*) combined with the indicated X-linked gene (*red*) in F3 parental cells (*upper panels*) or in the indicated XCI revertant clones (*lower panels*). The chromosomal position of the genes analysed is depicted on the diagram on the *left*. In F3 cells, active alleles of *Cybb*, *Ndufa1*, *Lamp2* and *Atp7a* are located away from the H3K27me3 coated inactive X indicating that these genes are expressed from the active X. In revertant clones 1#B and 1#F, active alleles of all four genes are located at the vicinity of the *Hprt1* signal marking the X^P^ and away from the H3K27me3 accumulation which indicates that these genes are transcribed from the active X. Moreover, *Cybb* and *Ndufa1* are also transcribed, in these clones, from the inactive X as illustrated by a faint signal at the periphery of H3K27me3 accumulation (*arrowheads*). Maximal projection after deconvolution. *Scale bar* 5 μm. **d** Cumulative histograms of the percentages of nuclei with the depicted expression pattern in F3 TS cells and in nuclei of 4 independent XCI revertant clones (analysis performed at passage 8 or later after clone picking). An *Hprt1* signal (*green* pinpoint) identifies the X^P^, while an H3K27me3 domain marks the inactive X (*yellow* domain). In XCI revertant clone 1#A, X-linked genes *Lamp2* and *Atp7a* that show high levels of H3K27me3 as assessed by ChIP-on-chip show a preferential expression from the reactivated X^P^. In contrast, X-linked genes *Cybb* and *Ndufa1* displaying low levels H3K27me3 by ChIP-on-chip show significant bi-allelic expression compared to F3 cells (*χ* ^2^ test *p* value <0.05). No significant difference is observed amongst XCI revertant clones (*χ* ^2^ test *p* value >0.05).  See Additional file 10 for probe position. *n* > 150

ChIP-chip analyses for H3K27me3 in male TS cells (F2 cell line) and in F3 and revertant clone 1#A female TS cells further specified that this histone mark preferentially associated with alleles on the inactive X that are expressed from the active X in TS cells as compared to intergenic regions or to X-linked genes that are silent in TS cells, as previously described in other cell types showing either R-XCI or I-XCI [20, 30] (see Additional file 3C, D, E). The level of H3K27me3 at expressed X-linked genes in I-XCI revertant 1#A was however significantly lower than H3K27me3 levels in the F3 parental cell line. Gene-by-gene clustering of H3K27me3 profiles in F2, F3 and clone 1#A revealed that this loss of H3K27me3 in 1#A cells compared to female F3 cells only occurs at ~ 12 % of expressed genes analysed, leading, at these genes, to an H3K27me3 percentage very similar to that observed in male F2 cells (Fig. 3a, b and see Additional file 4).

We then addressed whether the level of H3K27me3 in the gene bodies impacted gene transcription, using immuno-RNA-FISH. To specifically distinguish the X^P^, we analysed *Hprt1* transcription, whilst the transcription and the topological localisation of 4 X-linked loci (*Cybb*, *Ndufa1*, *Lamp2* and *Atp7a*) were compared to the accumulation of H3K27me3 on the inactive X (Fig. 3c, d). In nuclei of I-XCI revertant clones 1#A, 1#B, 2#B and 1#F, *Lamp2* and *Atp7a*, two genes that show levels of H3K27me3 similar to F3 female cells (Fig. 3a), appeared to be transcribed from the active X^P^ (i.e. lacking H3K27me3 and transcribing *Hprt1*^*WT*^) in the majority of nuclei. In contrast, the *Ndufa1* locus, which shows low levels of H3K27me3 in 1#A cells compared to F3 cells, exhibited significant bi-allelic expression in all revertant clones (20–30 % of nuclei). In addition, *Ndufa1* active maternal alleles lacked H3K27me3 staining and appeared located at the periphery of H3K27me3 accumulation on the inactive X. No *Cybb* signal could be detected in the majority of cells of XCI revertant clones (including 1#A cells) despite the fact that this gene displays low levels of H3K27me3 in these cells. Note that *Cybb* is poorly expressed in F3 cells (as measured by RNA-FISH) and in TS cells in general (as assessed by gene expression microarray; see Additional file 4 and [33]). However, in the few cells expressing *Cybb*, the gene was transcribed from both X-chromosomes in a higher percentage of I-XCI revertant clone cells compared to the F3 parental cells (Fig. 3c, d).

We also tested whether low levels of H3K27me3 at some X-linked genes of 1#A cells were associated with significant expression from both X-chromosomes using single-cell allelic RT-qPCR analysis. We measured the variability of expression levels of two H3K27me3-low genes, *Cox7b* and *Hmgn5*, amongst 1#A clonal cells (see Additional file 3F). *Cox7b* appeared significantly expressed from the X^M^—although at a lower level than from the X^P^—in a subset of 1#A cells. In contrast, no such extensive leak of expression was observed from the maternal *Hmgn5* allele on the same sample of cells.

These results indicate that the reversal of X-inactivation patterns is associated, at the vast majority of active X-linked genes, with an enrichment of H3K27me3 on the inactive X^M^. However, some specific genes resist H3K27me3 accumulation and are either expressed at low levels from the X^M^ in a subset of cells or repress the maternal allele using a mechanism different from H3K27me3 recruitment.

### Female TS cells transiently pass through a state in which the two X-chromosomes are active before switching X-inactivation profiles

**Fig. 4:**
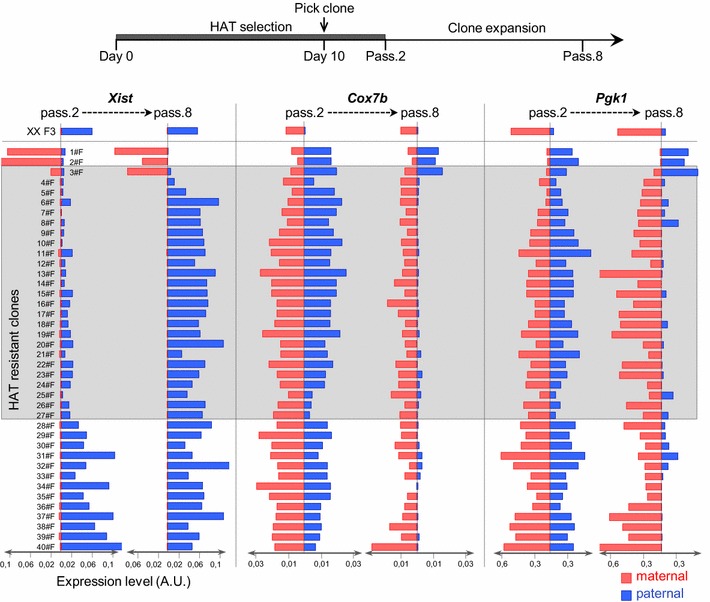
Global reactivation of the paternal X precedes the switching of X-inactivation profiles in female TS cells. Histograms showing the paternal and maternal expression levels of *Xist* and X-linked genes *Cox7b* and *Pgk1* in the indicated HAT-resistant clone assessed by allelic RT-qPCR. Two different time points during the selection procedure have been analysed: clones at passage 2 (pass.2) after clone picking (still under HAT selection) and the same clones after 6 additional passages without HAT (pass.8). Above the histograms, the diagram depicts the scheme of the HAT selection experiment used to isolate HAT-resistant clones analysed in this *panel*. Standardisation by *Rplp0* has been applied. *A.U.* Arbitrary Units

In order to understand how TS cells undergo this reversal of I-XCI, we analysed, by allelic RT-qPCR, the expression of *Xist* and of two other X-linked genes (*Cox7b* and *Pgk1*) at an earlier time point after clone isolation (in the presence of HAT selection). In the framework of the same HAT selection (experiment #F), at passage 2 after clone picking, three categories of clones could be distinguished (Fig. 4). Two out of 40 clones already showed an inverted I-XCI profile, 25 clones displayed low levels of *Xist* expression overall combined with a bi-allelic expression of both *Cox7b* and *Pgk1a*, while 13 clones exhibited a preferential paternal expression of *Xist* associated with strong bi-allelic expression of *Cox7b* and *Pgk1*. Since this latter expression profile evolves, after HAT release (passage 8), towards a return to a parental-like XCI pattern, these clones have probably locally and transiently reactivated both X^P^-linked loci together with the *Hprt1*^*WT*^ gene. As previously mentioned, similar transient, local and coordinated reactivations of several X-linked loci on the inactive X^P^ including *Hprt1*, *Cox7b* and *Pgk1a* have been observed in another TS cell line [20]. The fact that reactivation of specific X-linked genes takes place early during the selection process may indicate that the HAT treatment facilitates this phenomenon. We note, however, that these local relaxations of X-linked silencing do not seem to be sufficient—at least on their own—to trigger the reversal of I-XCI since none of these clones evolved towards a global X^P^ reactivation or X^M^ inactivation upon HAT release.

In contrast, robust bi-allelic expression of X-linked genes accompanied by low-level *Xist* expression in the second category of clones may indicate the presence of two-active X-chromosomes. In order to test this hypothesis, we launched a new HAT selection and prolonged the HAT treatment with the hope of maintaining the cells under this putative two-active-X state (Additional file 5A; experiment #C). Under these conditions, we recovered the same categories of HAT-resistant clones with similar frequencies as previously observed (see Fig. 1b). We then concentrated on three clones (1#C, 2#C and 3#C) that showed low levels of *Xist* expression under HAT pressure (passage 4 after clone picking) both in RT-qPCR and by *Xist* RNA-FISH (Additional file 5A, B). In these clones, we analysed the expression of 9 X-linked genes by allelic RT-qPCR at two different time points: passage 4 (under HAT) and passage 14 (i.e. 8 passages after HAT release). Coincidently with reduced *Xist* expression, a strong bi-allelic expression of most X-linked genes was observed in all three clones maintained in HAT. We noted, however, that *Rps6ka6* retained a preferential expression from the X^M^ in two clones, suggesting that the reactivation of paternal alleles of different X-linked genes may not occur completely synchronously in all clones. After HAT removal, 2 out of three clones evolved towards a maternal expression of X-linked genes associated with *Xist* paternal induction, while the third clone inactivated the maternal X-chromosome (Additional file 5A).

We conclude from these observations that the allelic switch of I-XCI patterns is probably mediated—at least in part—by a repression of paternal *Xist* expression associated with a global X^P^ reactivation, which leads to the presence of two-active X-chromosomes. We cannot, however, exclude that some TS cells directly switch from parental to inverted I-XCI upon HAT selection without transiting through a “two-active-X” state. From the two-active-X state, TS clones rapidly evolve, either towards a return to the parental-like configuration, or undergo I-XCI reversal.

### Allelic switch of I-XCI patterns does not require a transition through pluripotency

Only two mouse cell types are known to tolerate a lack of XCI: the primordial germ cells and the cells of the inner cell mass of the blastocyst. This particularity may be related to the pluripotency of these cells (for review see [9, 34] and [15]). We thus tested whether the presence of two-active X-chromosomes in HAT-resistant TS cells was associated with a conversion to a pluripotent state. To this end, we measured by RT-qPCR the expression level of pluripotency markers *Oct3/4* and *Nanog* in TS clones maintained under HAT and shown to harbour two active Xs. No significant expression of either marker could be detected in such clones either at the population level (see Additional file 6A) or at the single-cell level (see Additional file 6B and Additional file 7). These clones still expressed trophectoderm-specific markers (see Additional file 6C, D) thereby indicating that TS cells do not detectably change cell-type identity upon reversal of I-XCI.

### The choice of which X to inactivate after X^P^ reactivation occurs synchronously and homogeneously within clonal TS cell populations showing two-active X-chromosomes

**Fig. 5:**
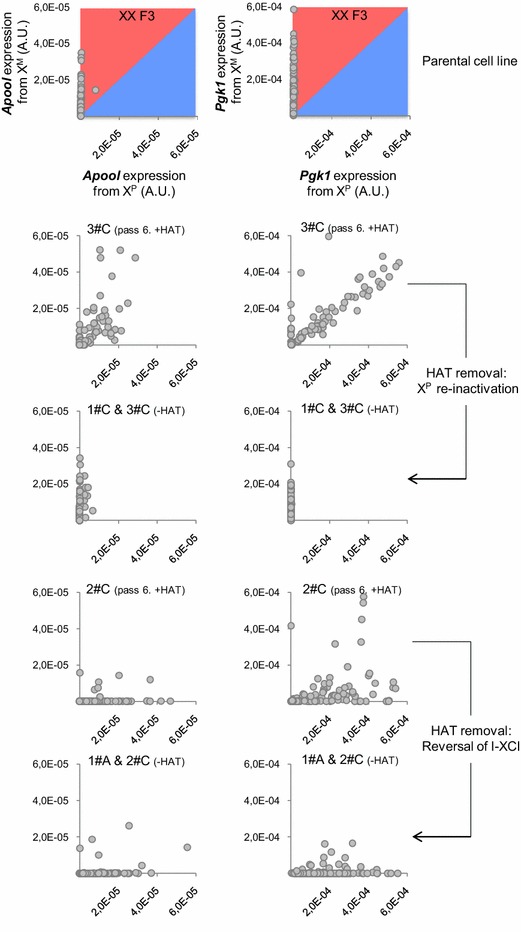
Biased X-inactivation occurs upon HAT release in clonal cell populations harbouring two-active X-chromosomes. Scatterplots of expression levels from the paternal (*x*-axis) relative to the maternal (*y*-axis) X-chromosome for the indicated gene in individual cells of the indicated clones assessed by allelic RT-qPCR (BioMark, Fluidigm). Each *dot* represents a single TS cell. (+HAT): cells at passage 6 maintained under HAT pressure; (−HAT): cells after HAT removal. The same samples of cells have been analysed for the expression of the genes shown in this figure and in Additional files 8 and 9. F3: *n* = 77; 3#C (+HAT): *n* = 75; 2#C (+HAT): *n* = 108; 1#C & 3#C (−HAT): *n* = 144; 1#A & 2#C (−HAT): *n* = 141. (See Additional file 7) for raw quantifications. *A.U.* Arbitrary Units

Theoretically, from the two-active-X state, individual TS cells may either choose to inactivate the X^P^ or the X^M^. Under the hypothesis of a random, cell-autonomous, choice, we expect to observe mosaic clones consisting of cells of each configuration in number almost equal. Our results on TS clones after HAT release rather suggest a strong bias towards one or the other configuration depending on the clone. In order to determine precisely the mosaicism of clonal TS cell populations, we quantified the expression of *Xist* and of 5 other X-linked genes, at the single-cell level, using allelic RT-qPCR in 4 different clones: 2 clones showing a parental-like I-XCI (1#C & 3#C) and 2 clones showing an inverted I-XCI (1#A & 2#C) after HAT removal (−HAT) (Fig. 5 and Additional files 7, 8 and 9). In agreement with our previous results on clonal cell populations, the vast majority of cells of clone 1#C and clone 3#C showed a homogeneous XCI profile with a preferential expression of maternal alleles of the genes we tested. The situation is less clear in clone 1#A and clone 2#C showing a switch of I-XCI in which we observed, depending on the gene, a subset of cells displaying a bi-allelic expression (i.e. *Atp7a, Cox7b* and *Pgk1*). Importantly, we did not observe, within a given clone, cells with the opposite XCI profile than the predominant configuration.

When we performed the same single-cell analysis on one clone of each category maintained under HAT pressure (3#C and 2#C (+HAT)), we observed low levels of *Xist* expression (see Additional file 8) associated with bi-allelic expression of most X-linked genes in most cells of clone 3#C confirming the presence of two-active X-chromosomes in those cells (Fig. 5, Additional file 9 and Additional file 7 for quantification results). In contrast, cells of clone 2#C maintained under HAT pressure already showed a significant maternal *Xist* expression in 51 % of the cells (see Additional file 8) accompanied by a heterogeneous behaviour amongst X-linked genes with some genes being bi-allelically expressed in most cells (*Atp7a*, *Cox7b*), while others already showed a predominant paternal expression (*Apool*, *Sh3bgrl*) (Fig. 5 and Additional file 9). This suggests that the switch of XCI patterns is not synchronously established in all the cells of a given clone and at all X-linked genes of a given cell. Of note, we did not detect any marked cell death or higher levels of spontaneous differentiation during clone expansion that may be suggestive of a counter-selection or of a growth disadvantage of a subset of cells within clones.

In conclusion, the secondary biassed XCI we have observed here implies that the choice of which X to re-inactivate in this cellular system of TS cells selected with HAT does not involve the same mechanism as the random choice of active and inactive X-chromosomes occurring upon ES cell and ICM differentiation.

## Discussion

Here, we show that trophoblast stem cells derived from the polar trophectoderm exhibit a remarkable plasticity allowing them to reactivate the paternal X and to almost-fully invert I-XCI profiles (see Additional file 11A). Similar X^P^ reactivation or reversal of I-XCI had already been observed in vivo in mutant contexts in which *Xist* regulation had been impaired [26, 35]. Our results further suggest that trophoblast cells can undergo this allelic switch in the absence of experimentally induced *Xist* deregulation. This plasticity of I-XCI in the trophectoderm may serve as a back-up mechanism allowing placental progenitors to promptly adapt to mutations affecting genes on the maternal X or to environmental pressure favouring the usage of one allelic form compared to the other, like in the present study, in which only the cells expressing the paternal *Hprt1* allele can survive in a HAT-containing medium. In the case of the present study, it is also possible that the HAT treatment represents a form of stress for the cells that may modify some layers of epigenetic regulation thereby facilitating the changes of XCI profiles. More generally, the plasticity of I-XCI may also be at work in the case of dysfunction of X^P^ silencing in order to maintain a single dose of X-linked gene products. It may also be responsible for inactivating one of the two maternal Xs in X^M^X^M^ parthenogenetic embryos [1, 36, 37] (see Additional file 11B).

To undergo the switch of I-XCI profiles, some—if not all—TS cells globally reactivate their paternal X to reach a two-active-X state. This transition correlates with a reversal of epigenetic mechanisms maintaining the inactive state including a loss of *Xist* coating and a resetting of histone modifications specific to the inactive X such as H3K27me3. The two-active-X state appears extremely labile indicating that TS cells cannot stand a double dose of X-linked gene expression for multiple cell divisions. Interestingly, the EED component of the PRC2 complex—the complex responsible for adding and maintaining H3K27me3 on the inactive X—becomes necessary to maintain X^P^ silencing only after TS cell differentiation [38, 39]. This suggests that the silencing of the paternal X is not fully stabilised in undifferentiated TS cells which may facilitate the reactivation of this chromosome and results in the presence, at a low frequency, of TS cells exhibiting active paternal alleles among the global TS cell population. Alternatively and not exclusively, the *Kdm6a/Utx* X-linked gene, which escapes from X-inactivation and encodes an H3K27me3 demethylase, may be responsible for the H3K27 demethylation of the paternal X which accompanies the reversal of I-XCI profiles in TS cells. Indeed, *Kdm6a/Utx* has been shown to be necessary for somatic cell reprogramming towards pluripotency [40], which is accompanied by a reactivation of the inactive X, and H3K27me3 demethylase activities participate in the repression of *Xist* in ES cells [41].

Collectively, these features evoke the dynamic turnover—erasure and de novo establishment—of methylation marks used in human ES cells to maintain DNA methylation profiles, which leads to ES cell populations showing heterogeneous methylation patterns. In contrast, in differentiated cells, methylation marks are stably maintained through copying them from mother to daughter cells, a mechanism that is thought to be more vulnerable to the insertion and to the propagation of aberrant methylation marks in the cell population [42]. Following this reasoning, one may speculate that the ease of reactivation of X^P^ alleles in undifferentiated TS cells may be permitted by similar ongoing turnover of epigenetic marks such as H3K27me3.

From the two-active-X metastable state, the cells evolve either towards a return to the parental profile or towards a switch of I-XCI profiles. Unexpectedly, our analysis shows that within clonal TS cell populations displaying two active Xs, sister cells homogeneously choose the same inactivation path as illustrated by the lack of mosaic clones comprising cells that have inactivated the X^P^ mixed with cells that, conversely, have silenced the X^M^ (see Additional file 11B). The choice of which X to inactivate after X^P^ reactivation in TS cells therefore differs from the random choice mechanism recruited during ES cell differentiation. In addition, the process of X^P^ reactivation followed by X^P^ re-inactivation occurs synchronously at most X-linked genes and between sister cells, while the de novo inactivation of the X^M^ appears more chaotic and is more rapidly established. Furthermore, while X^P^ re-inactivation is only observed after HAT removal, X^M^ silencing may take place under HAT pressure. These results suggest that clones returning to the parental profile have retained a memory of the inactive state that inhibits I-XCI reversal and may explain why I-XCI reversal occurs less frequently. This is reminiscent of the memory of the inactive state observed in cells of the trophoblast lineage of cloned embryos originating from somatic cell nuclear transfer [43]. In contrast, the stochastic behaviour of TS cells showing a switch of I-XCI supports the hypothesis that the maternal imprint that presumably prevents X^M^ inactivation has been erased—at least partially—in these clones and that the HAT pressure favours the inactivation of the X^P^. In these cells, some genes on the X^M^ however escape both from de novo H3K27trimethylation and from X-wide silencing indicating that they may be refractory to inactivation or that a tight control of their expression is not crucial for TS cell homeostasis. Alternatively, these non-cell-autonomous behaviours suggest cell-to-cell communications within clonal cell populations.

## Conclusions

In summary, TS cells appear to be able to reprogram X-chromosome expression back and forth without any detectable change in cell-type identity. Notably, the reactivation of the paternal X is not associated with a conversion to a pluripotent state as opposed to reactivation of the inactive X in the embryonic lineage, in the germline, or during somatic cell reprogramming into iPS cells. This suggests that these stem cells show unique epigenomic features facilitating the reprogramming of gene expressions and thereby enabling trophoblast stem cells to adapt to any impairment of programmed mono-allelic gene expression that may occur during extraembryonic development. Such specific features put forward the TS cell model as an alternative background to explore lineage-specific determinants of genome plasticity/multipotency.

## Methods

### TS cell culture and HAT/6-TG treatments

Male F2 and female F3 TS cell lines used in this study were derived in the lab and have been previously described [12, 13]. F3 cells carry an X^P^ from 129/Sv origin and an X^M^ from 129.Pgk1a origin providing polymorphisms allowing for allelic distinction. TS culture conditions were as previously described [12, 13, 44]. Cells were pre-treated or not with 30 μM of 6-TG (Sigma A4882) for a week then treated with HAT (100 μM Hypoxanthine, 0.4 mM Aminopterin, 16 mM Thymidine) for 10 days. Cells were then grown for two additional passages in medium containing HT but lacking Aminopterin, which is the component assimilated by HPRT, to allow TS cells to recover from the selection before they are grown in standard TS medium again. The frequency of HAT-resistant cells within the framework of a given HAT selection corresponds to the number of HAT-resistant clones observed at the end of the selection divided by the number of TS cells initially plated. The average frequency of HAT resistance corresponds to the mean of frequencies observed in the time course of independent selection experiments (#A, #B, #C, #D, #E and #F).

### ChIP-chip procedure and data analysis

ChIP assays were performed as previously described [45] using an H3K27me3 antibody (07-449 from EMD Millipore, Billerica, MA, USA) and analysed as described previously [19, 20]. ChIP-chip data have been deposited under Gene Expression Omnibus accession number [GSE:68536]. Two replicates of each experiment are available and show similar results.

Raw data were analysed using the Tobias Straub protocol (http://www.epigenesys.eu/images/stories/protocols/pdf/20111025114444_p43.pdf) with the Bioconductor R interface. This protocol includes quality assessment, data normalisation and transformation. The gene expression data of TS cells were extracted from [GSE:15519] [33].

### RNA-FISH and Immuno-RNA-FISH

RNA-FISH and immuno-RNA-FISH procedures were carried out as described in [46] (H3K27me3 antibody, 07-449; EMD Millipore). For details of our experimental procedures, for probe localisation and for image capture protocol, see [19].

### Single-cell gene expression analysis

Single-cell gene expression analysis was performed as described previously in [19, 20] and as recommended by Fluidigm (http://www.fluidigm.com/single-cell-expression.html; South San Francisco, CA, USA). Briefly, TS cells were sorted by fluorescence-activated cell sorting using the MoFlo system (Beckman Coulter, Brea, CA, USA), and individual cells were distributed into wells or 96-well plates containing 5 μl of CellsDirect resuspension buffer (Invitrogen, Carlsbad, CA, USA). The preamplification step consisted of 20 cycles using a mix of universal primer pairs to preamplify each gene simultaneously. Preamplification was followed by exonuclease I treatment (New England Biolabs, Ipswich, MA, USA), and allelic qPCR was performed on a BioMark thermal cycler (Fluidigm). Raw efficiencies of each PCR assay and allelic specificity were measured on control DNA within each experiment. Transcript levels were extrapolated using the raw PCR efficiencies, thus allowing the direct comparison of different genes. Controls for allelic specificities of PCR assays are available upon request. Three house-keeping control RNAs (*Gapdh*, *Hist2h2a* and *Rplp0*) were systematically quantified in parallel in each single cell. See Additional file 7 for raw quantifications. Detailed analyses and statistical tests have been performed using the Qlucore Omics Explorer 2.3 (QLUCORE Company). For primer sequences, see [19].

## Additional files

10.1186/s13072-015-0044-2 HAT resistant clones of F3 cells are *bona fide* TS cells indiscernible from the parental cell line. **A**. Representative pictures of F3 TS cells before HAT treatment and of 4 independent HAT resistant clones showing different profiles of *Xist* expression. Each individual clone is identified by a number and a capital letter which refers to the selection experiment from which the clone has been isolated (see Fig. 1 for experiment description). **B.** Cumulative histograms showing the level of expression of TS specific markers *Cdx2* and *Eomes* and of markers of TS differentiated states, *Esx1* and *Prl3b1* [19], assessed by RT-qPCR on a representative sampling of HAT resistant clones obtained during different HAT selections (analysis performed at passage 8 or later after clone picking). Levels have been standardised by *Rplp0*. Levels measured in F3 TS cells and in F3 TS cells differentiated for 5 days are shown for comparison. For F3 parental female cells, mean and standard deviation have been calculated on the basis of three independent cell cultures and differentiation experiments. A.U. Arbitrary Units. Log scale is used.
10.1186/s13072-015-0044-2 X-linked gene expression profiles in various types of HAT resistant clones. **A.** RNA-FISH for *Xist* (red) in some of the clones depicted in Fig. 1C. Representative images and cumulative histrograms of the percentage of nuclei with the depicted expression pattern are shown (analysis performed at passage 8 or later after clone picking). No significant difference is observed between any of the clones and F3 parental cells (χ^2^ test). n > 100. Scale bar = 5 μm. **B.** Allelic RT-qPCR analysis of X-linked gene expression in HAT resistant clones showing an F3-like expression profile (analysis performed at passage 8 or later after clone picking). Cumulative histograms showing the paternal (blue) and maternal (red) expression levels of the indicated X-linked are shown. Standardisation by *Rplp0* has been applied. For F3 parental female cells, mean and standard deviation have been calculated on the basis of three independent cell cultures. The chromosome diagram on the left shows the localisation of the genes analysed in RT-qPCR and by RNA-FISH in panel **B**, **C** and **D**. **C.** Cumulative histograms of the percentages of nuclei with the depicted RNA-FISH expression pattern in F3 TS cells and in nuclei of 3 independent HAT resistant clones showing a parental-like *Xist* expression profile (analysis performed at passage 8 or later after clone picking). Seven different X-linked genes distributed along the X-chromosome have been analysed (see Additional file 10 for probe coordinates). Representative images of double RNA-FISH for *Xist* and for the indicated X-linked gene in clones 12#F and 23#F are shown on the right hand side of the histogram. The inactive X chromosome is identified through *Xist* RNA accumulation (red). Primary transcription at the indicated X-linked gene is co-detected in green. < 1 % of F3 parental TS cells show an *Hprt1* signal in agreement with the inactivation of the wild type, paternal, *Hprt1* allele. In contrast, an *Hprt1* signal associated with the *Xist* domain is detected in 25 to 50 % of nuclei of HAT resistant clones 12#F, 23#F and 36#F indicating that these clones have locally reactivated the *Hprt1* ^*WT*^ allele on the inactive paternal X. Note that active *Hprt1* alleles appear to extrude from the *Xist* domain in these category of HAT resistant clones. No significant difference in the expression profiles is observed between any of the clones and F3 parental cells at X-linked genes *Tfe3*, *Lamp2*, *Atp7a*, *Pgk1*, *Fgd1* and the X-inactivation escaper *Kdm5c* (χ^2^ test, *p* value > 0.05). n > 150. Scale bar = 5 μm. **D.** Allelic RT-qPCR analysis of *Cox7b* and *Atp7a* in 8 clones showing an allelic inversion of *Xist* expression profiles (analysis performed at passage 8 or later after clone picking). Cumulative histograms showing the paternal (blue) and maternal (red) expression levels of the indicated X-linked are shown. Standardisation by *Rplp0* has been applied. For F3 parental female cells, mean and standard deviation have been calculated on the basis of three independent cell cultures.
10.1186/s13072-015-0044-2 H3K27me3 accumulates on the inactive X chromosome in I-XCI revertant clones. **A.** Representative image of immunostaining followed by RNA-FISH for H3K27me3 (green) and *Xist* (red) on female F3 TS cells and on cells of 2 I-XCI revertant clones. Scale bar = 5 μm. **B.** Cumulative histogram showing the percentage of nuclei exhibiting an accumulation of *Xist* RNA only (red), co-accumulation of *Xist* RNA and H3K27me3 (yellow) and accumulation of H3K27me3 only (green) in F3 TS cells and in cells of I-XCI revertant clones 1#A and 1#C (analysis performed at passage 8 or later after clone picking). No significant difference is observed between any of the clones and F3 parental cells (χ^2^ test). n > 100. **C.** Boxplots of the distribution of H3K27me3 along expressed X-linked genes in male F2 TS cell, in F3 female TS cells and in cells of I-XCI revertant clone 1#A (analysis performed at passage 8 or later after clone picking)(see Additional file 4 for gene by gene percentage of enrichment). Expression data were extracted from the Gene Expression Omnibus database [GSE:15519] [33]. *n* = 203 expressed X-linked genes. **p*-*value* < 0.05 by Kolmogorov–Smirnov test. **D.** Boxplots of the distribution of H3K27me3 along X-linked intergenic probes. *n* = 263245 intergenic probes. No significant difference between the 3 cell lines was observed (Kolmogorov–Smirnov test). **E.** Boxplots of the distribution of H3K27me3 along the body of X-linked genes that are not significantly expressed in TS cells. Expression data were extracted from the Gene Expression Omnibus database [GSE:15519] [33]. *n* = 292 X-linked genes. No significant difference between the 3 cell lines was observed (Kolmogorov–Smirnov test). **F.** Scatterplot of expression levels from the paternal (x-axis) relative to the maternal (y-axis) X chromosome for the indicated gene in 56 individual cells of the 1#A I-XCI revertant clone assessed by allelic RT-qPCR (analysis performed at passage 8 or later after clone picking). Each dot represents a single TS cell. Similar results have been obtained on another I-XCI revertant clone. Expression levels have been normalised by the median value for each gene. A.U.: Arbitrary Units.
10.1186/s13072-015-0044-2 ChIP-on-chip analysis of H3K27me3 distribution along X-linked genes in male F2 TS cells, in female F3 TS cells and in I-XCI revertant cells from clone 1#A. Mean of two replicate ChIP experiments.
10.1186/s13072-015-0044-2 Global reactivation of the paternal X precedes the switching X-inactivation profiles in female TS cells. **A.** Cumulative histograms showing the expression levels of paternal (blue) and maternal (red) alleles of the indicated gene, assessed by allelic RT-qPCR, in parental F3 cells and in 3 independent clones showing low levels of *Xist* RNA. Two time points have been analysed: clones at passage 4 after clone picking and still under HAT pressure (Pass.4 histograms) and the same clones, 8 passages after HAT release (Pass.14 histograms). Note that, in contrast with other X-linked genes, *Pbdc1* maintains a bi-allelic expression after HAT release in agreement with its status of XCI escaper. Standardisation by *Rplp0* followed by standardisation by the median value for each gene have been applied to allow direct comparison of the different genes on the same histogram. For F3 parental female cells, mean and standard deviation have been calculated on the basis of three independent cell cultures. Above the histograms, the diagram depicts the scheme of the HAT selection experiment used to isolate HAT resistant clones analysed in this panel. A.U. : Arbitrary Units. **B.** Representative image of *Xist* RNA-FISH on nuclei of clone 2#C and clone 3#C maintained under HAT pressure. On the right, the histogram shows the percentages of nuclei of each depicted category. Clones under HAT pressure harbour significantly different *Xist* expression profiles compared to F3 parental cells (χ^2^ test p-value < 0.05). n > 150.
10.1186/s13072-015-0044-2 The two-active-X state of early HAT resistant clones is not associated with cell reprogramming towards pluripotency. **A.** Cumulative histograms showing the expression levels of pluripotency markers *Nanog* and *Oct3*/4 measured by RT-qPCR in F3 parental female TS cells and in 3 clones harbouring two active X-chromosomes. Expression levels in XX ES cells (LF2 cell line) are shown for comparison. Standardisation by *Rplp0* has been applied. A.U.: Arbitrary Units. **B.** Same as in panel **A** on individual cells of each population. Small horizontal bars represent median values. See Additional file 7 for raw quantifications. **C.** Cumulative histograms showing the level of expression of TS specific markers *Cdx2* and *Eomes* and of markers of TS differentiated states, *Esx1* and *Prl3b1* [19], assessed by RT-qPCR on a the same clones as in panel **A**. Expression levels have been standardised by *Rplp0*. Expression levels measured in F3 TS cells and in F3 TS cells differentiated for 5 days are shown for comparison. A.U. Arbitrary Units. Log scale is used. **D.** Same as in panel **C** for *Cdx2* and *Eomes* on individual cells of each population. Small horizontal bars represent median values. See Additional file 7 for raw quantifications.
10.1186/s13072-015-0044-2 Single-cell RT-qPCR analysis in F3 parental cells, in cells of clones 1#C and 1#A after HAT removal and in cells of clones 3#C and 2#C both before and after HAT release.
10.1186/s13072-015-0044-2 Single-cell analysis of *Xist* expression in clonal cell populations under HAT selection and after HAT removal. Scatter-plots of *Xist* expression levels from the paternal (x-axis) relative to the maternal (y-axis) X chromosome in individual cells of the indicated clones assessed by allelic RT-qPCR (BioMark, Fluidigm). Each dot represents a single TS cell. (+HAT): cells at passage 6 maintained under HAT pressure; (-HAT): cells after HAT removal. The same samples of cells have been analysed for the expression of the genes shown in Fig. 5 and in Additional file 9. For clones showing parental-like profiles or inverted I-XCI after HAT removal, cells from two different clones are shown. No significant difference was detected between cells of these two clones (Kolmogorov–Smirnov test). In clone 3#C under HAT pressure, one cells out of 74 tested show a significant *Xist* expression (> 0.5 10^−5^ A.U. corresponding to level of *Xist* RNA molecules in a *Xist* domain), while, in clone 2#C under HAT pressure, 55 cells out of 108 tested show a significant *Xist* expression. F3: n = 77; 3#C (+HAT): n = 75; 2#C (+HAT): n = 108; 1#C & 3#C (-HAT): n = 144; 1#A & 2#C (-HAT): n = 141. An additional table shows the raw quantification results (see Additional file 7). A.U.: Arbitrary Units.
10.1186/s13072-015-0044-2 Single-cell analysis of gene expression of X-linked gene *Atp7a*, *Cox7b* and *Sh3bgrl* in clonal cell populations under HAT selection and after HAT removal. For details (see Additional file 8). See Additional file7 for raw quantifications.
10.1186/s13072-015-0044-2 Name and chromosomal position of X-linked RNA-FISH probes used in the study.
10.1186/s13072-015-0044-2 Summary of the metastable states of I-XCI in female TS cells observed in the present study. **A.** Table summarising the characteristics of the various TS cell populations observed in the study. **B.** We observe in the present study that undifferentiated TS cells spontaneously reactivate the paternal X partially or completely at a low frequency. Under specific environmental cues (including, in the case of the present study, the HAT selection) or mutation that either prevent X^P^ inactivation or favour the expression of X^P^ genes, TS cells carrying two active X chromosomes may undergo *de novo* inactivation of the maternal X.

## Abbreviations

X^M^: maternal X chromosome
X^P^: paternal X chromosome
TS cells: trophoblast stem cells
ES cells: embryonic stem cells
XEN cells: extraembryonic endoderm stem cells
ICM: inner cell mass
I-XCI: imprinted X-chromosome inactivation
R-XCI: random X-chromosome inactivation
HAT: Hypoxanthine, Aminopterin, Thymidine
RNA-FISH: fluorescence in situ hybridisation on ribonucleic acid
RT-qPCR: reverse transcription followed by quantitative polymerase chain reaction
ChIP: chromatin immuno precipitation
