# Supplementary figures and images for: A rapid passage through a two-active-X-chromosome state accompanies the switch of imprinted X-inactivation patterns in mouse trophoblast stem cells
Source: Epigenetics Chromatin. 2015 Dec 1;8:52. doi: 10.1186/s13072-015-0044-2 (PMC4665903; doi:10.1186/s13072-015-0044-2)

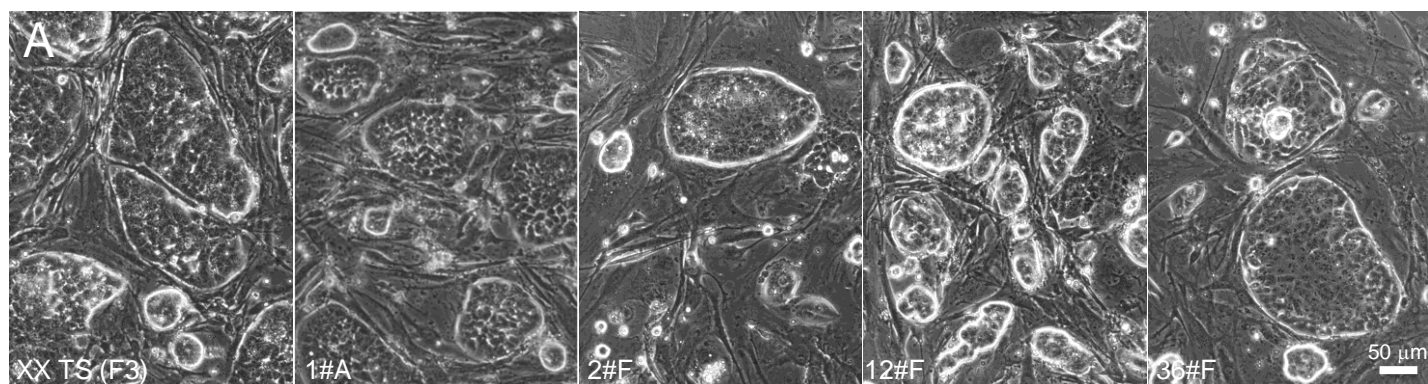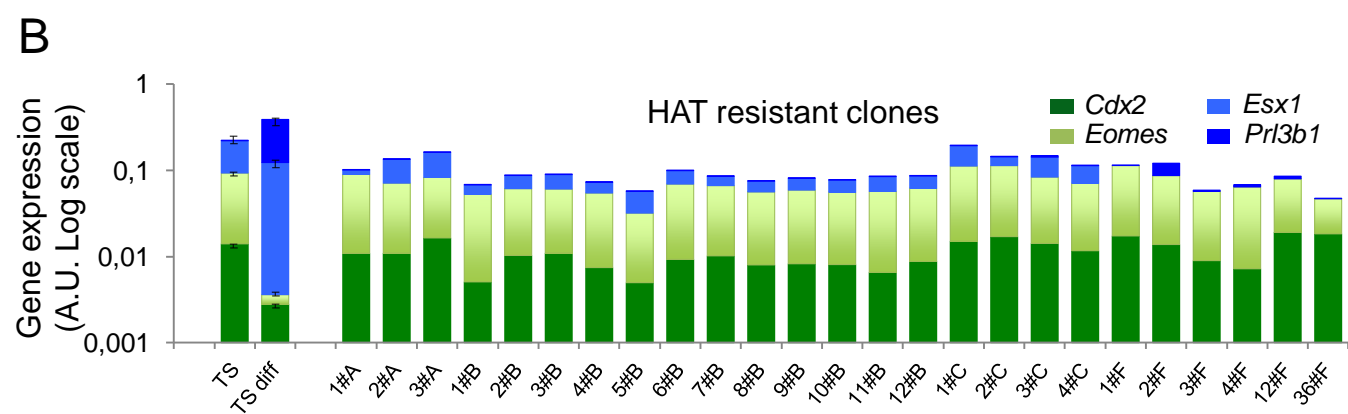

Supplement: Supplementary file 1 — 10.1186/s13072-015-0044-2 HAT resistant clones of F3 cells are bona fide TS cells indiscernible from the parental cell line. A. Representative pictures of F3 TS cells before HAT treatment and of 4 independent HAT resistant clones showing different profiles of Xist expression. Each individual clone is identified by a number and a capital letter which refers to the selection experiment from which the clone has been isolated (see Fig. 1 for experiment description). B. Cumulative histograms showing the level of expression of TS specific markers Cdx2 and Eomes and of markers of TS differentiated states, Esx1 and Prl3b1 [19], assessed by RT-qPCR on a representative sampling of HAT resistant clones obtained during different HAT selections (analysis performed at passage 8 or later after clone picking). Levels have been standardised by Rplp0. Levels measured in F3 TS cells and in F3 TS cells differentiated for 5 days are shown for comparison. For F3 parental female cells, mean and standard deviation have been calculated on the basis of three independent cell cultures and differentiation experiments. A.U. Arbitrary Units. Log scale is used. [file 13072_2015_44_MOESM1_ESM.pdf]

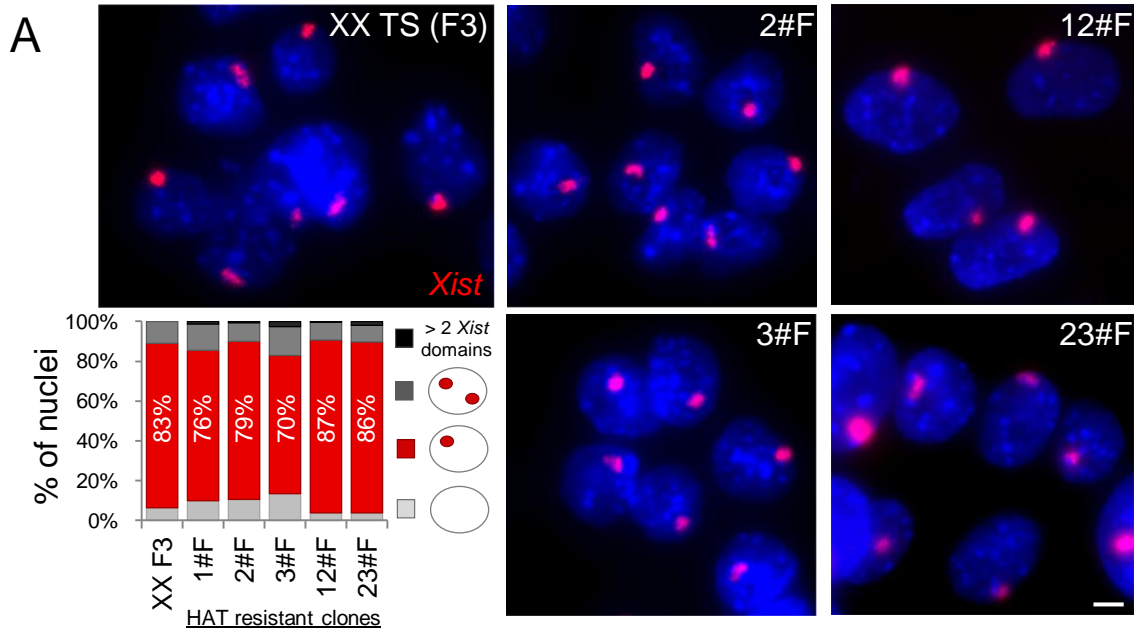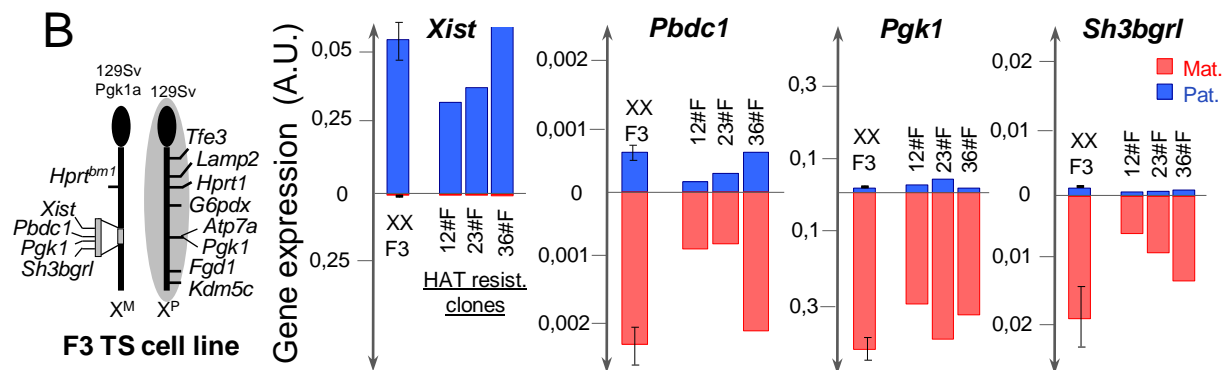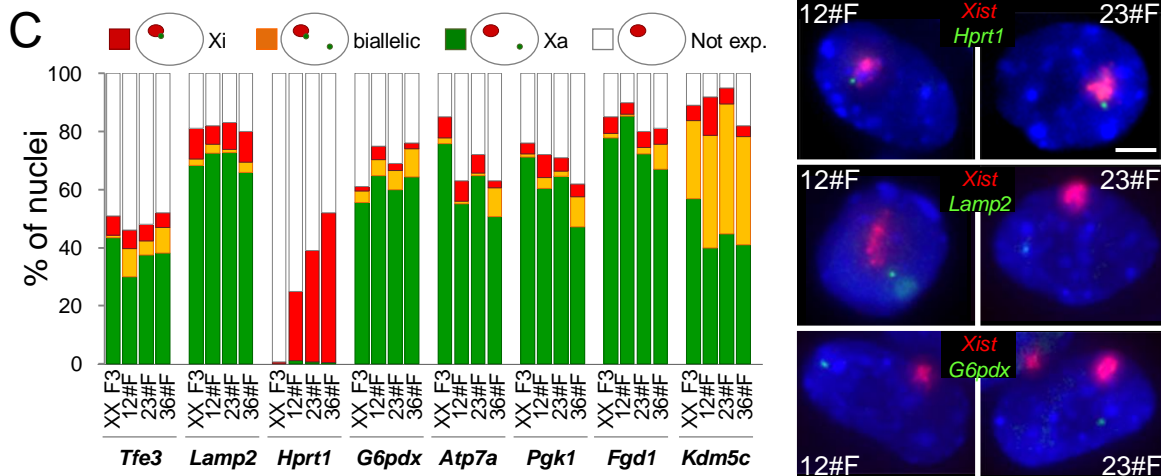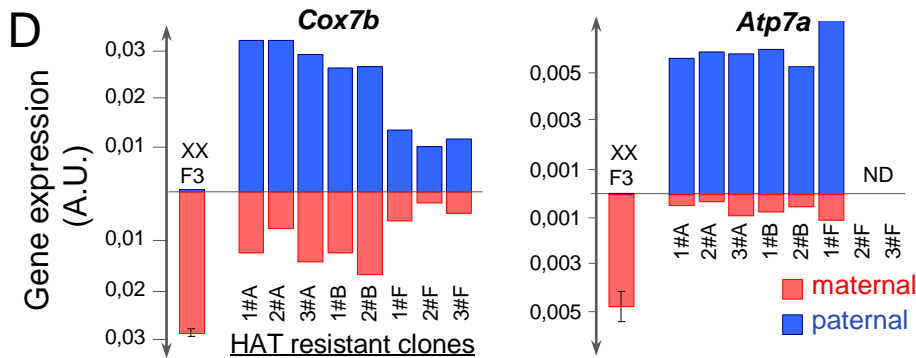

Supplement: Supplementary file 2 — 10.1186/s13072-015-0044-2 X-linked gene expression profiles in various types of HAT resistant clones. A. RNA-FISH for Xist (red) in some of the clones depicted in Fig. 1C. Representative images and cumulative histrograms of the percentage of nuclei with the depicted expression pattern are shown (analysis performed at passage 8 or later after clone picking). No significant difference is observed between any of the clones and F3 parental cells (χ2 test). n > 100. Scale bar = 5 μm. B. Allelic RT-qPCR analysis of X-linked gene expression in HAT resistant clones showing an F3-like expression profile (analysis performed at passage 8 or later after clone picking). Cumulative histograms showing the paternal (blue) and maternal (red) expression levels of the indicated X-linked are shown. Standardisation by Rplp0 has been applied. For F3 parental female cells, mean and standard deviation have been calculated on the basis of three independent cell cultures. The chromosome diagram on the left shows the localisation of the genes analysed in RT-qPCR and by RNA-FISH in panel B, C and D. C. Cumulative histograms of the percentages of nuclei with the depicted RNA-FISH expression pattern in F3 TS cells and in nuclei of 3 independent HAT resistant clones showing a parental-like Xist expression profile (analysis performed at passage 8 or later after clone picking). Seven different X-linked genes distributed along the X-chromosome have been analysed (see Additional file 10 for probe coordinates). Representative images of double RNA-FISH for Xist and for the indicated X-linked gene in clones 12#F and 23#F are shown on the right hand side of the histogram. The inactive X chromosome is identified through Xist RNA accumulation (red). Primary transcription at the indicated X-linked gene is co-detected in green. < 1 % of F3 parental TS cells show an Hprt1 signal in agreement with the inactivation of the wild type, paternal, Hprt1 allele. In contrast, an Hprt1 signal associated with the Xist d [file 13072_2015_44_MOESM2_ESM.pdf]

**A**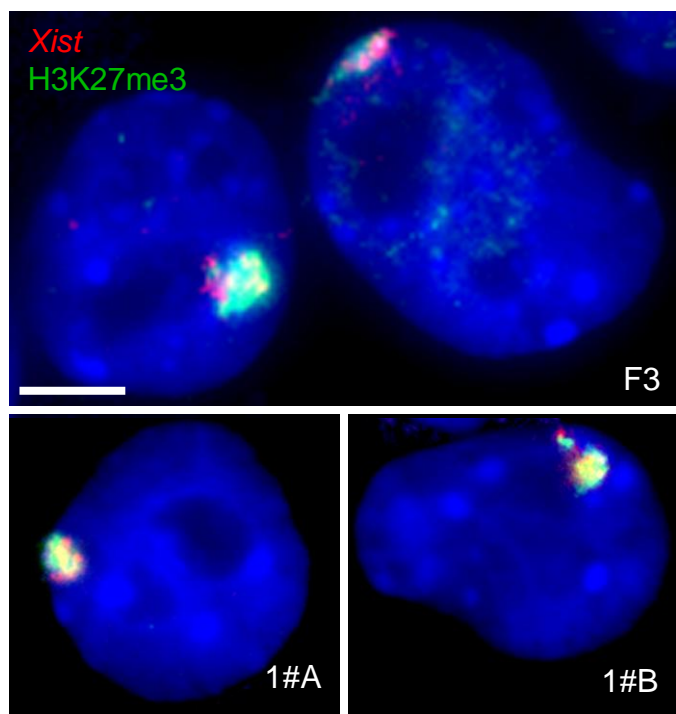**B**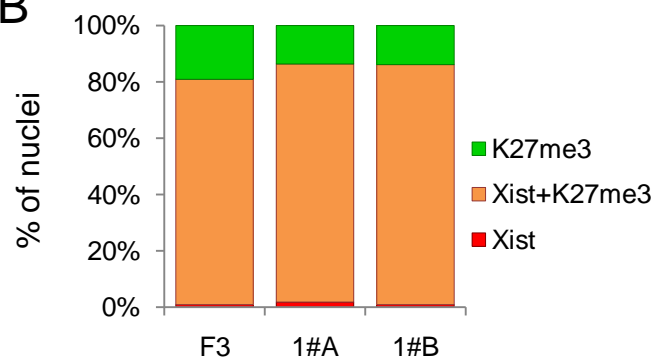**C**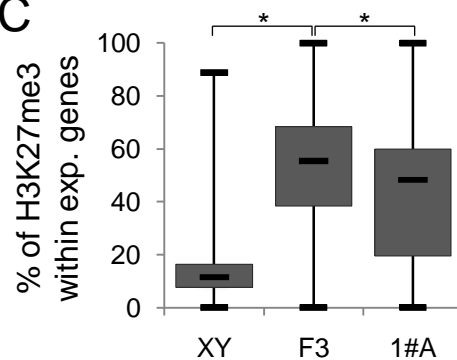**D**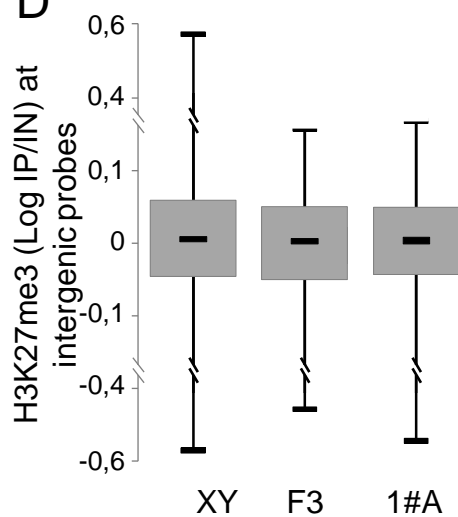**E**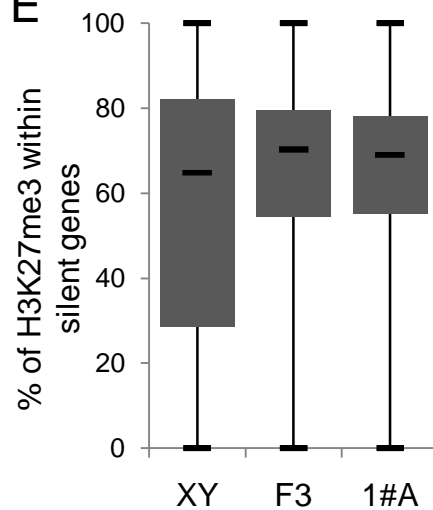**F**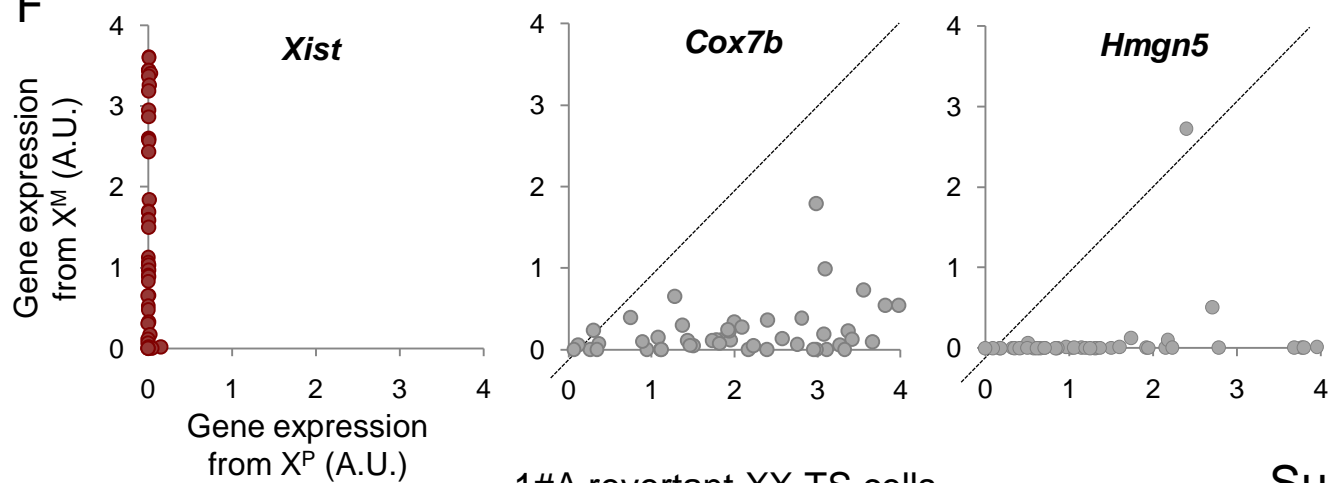

Supplement: Supplementary file 3 — 10.1186/s13072-015-0044-2 H3K27me3 accumulates on the inactive X chromosome in I-XCI revertant clones. A. Representative image of immunostaining followed by RNA-FISH for H3K27me3 (green) and Xist (red) on female F3 TS cells and on cells of 2 I-XCI revertant clones. Scale bar = 5 μm. B. Cumulative histogram showing the percentage of nuclei exhibiting an accumulation of Xist RNA only (red), co-accumulation of Xist RNA and H3K27me3 (yellow) and accumulation of H3K27me3 only (green) in F3 TS cells and in cells of I-XCI revertant clones 1#A and 1#C (analysis performed at passage 8 or later after clone picking). No significant difference is observed between any of the clones and F3 parental cells (χ2 test). n > 100. C. Boxplots of the distribution of H3K27me3 along expressed X-linked genes in male F2 TS cell, in F3 female TS cells and in cells of I-XCI revertant clone 1#A (analysis performed at passage 8 or later after clone picking)(see Additional file 4 for gene by gene percentage of enrichment). Expression data were extracted from the Gene Expression Omnibus database [GSE:15519] [33]. n = 203 expressed X-linked genes. *p-value < 0.05 by Kolmogorov–Smirnov test. D. Boxplots of the distribution of H3K27me3 along X-linked intergenic probes. n = 263245 intergenic probes. No significant difference between the 3 cell lines was observed (Kolmogorov–Smirnov test). E. Boxplots of the distribution of H3K27me3 along the body of X-linked genes that are not significantly expressed in TS cells. Expression data were extracted from the Gene Expression Omnibus database [GSE:15519] [33]. n = 292 X-linked genes. No significant difference between the 3 cell lines was observed (Kolmogorov–Smirnov test). F. Scatterplot of expression levels from the paternal (x-axis) relative to the maternal (y-axis) X chromosome for the indicated gene in 56 individual cells of the 1#A I-XCI revertant clone assessed by allelic RT-qPCR (analysis performed at passage 8 or later after clone picking). Each dot r [file 13072_2015_44_MOESM3_ESM.pdf]

A

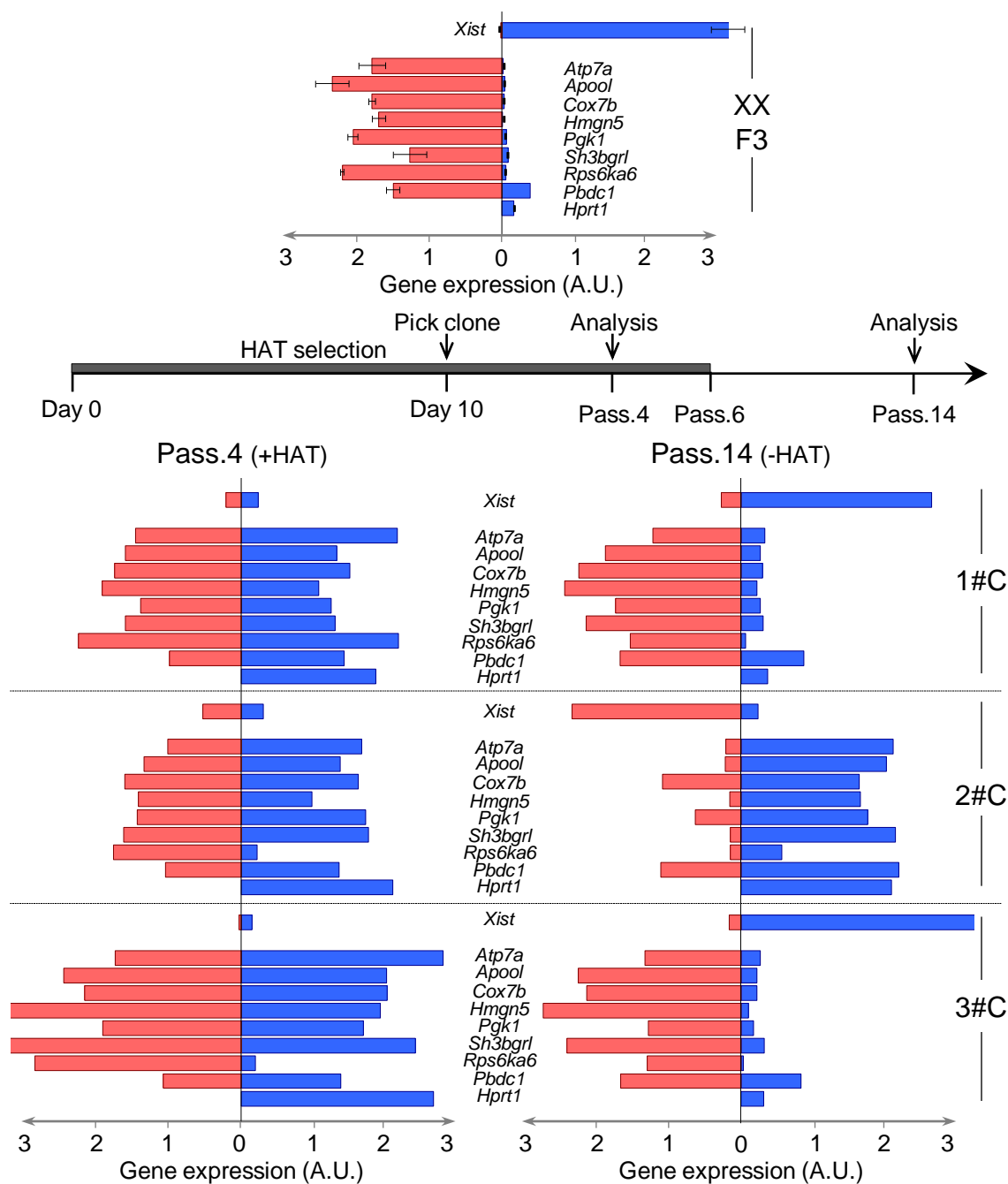

B

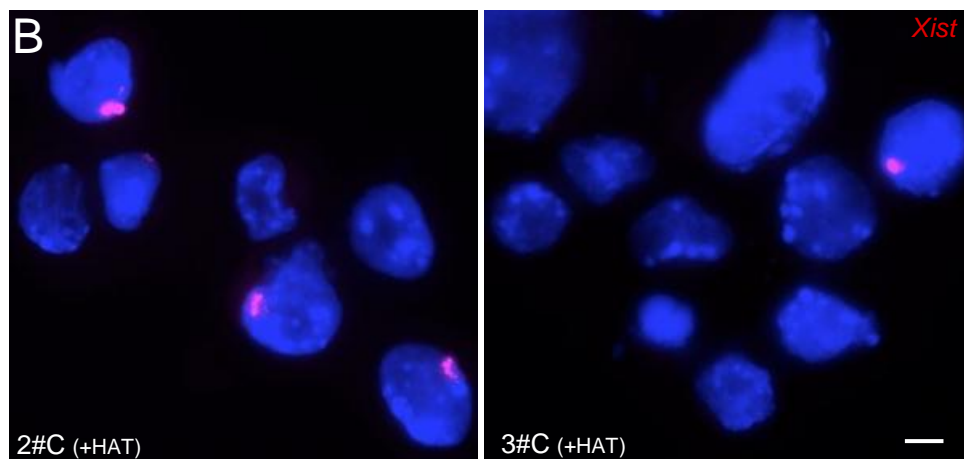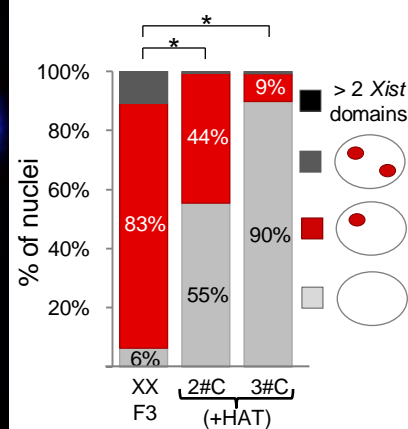

Sup.5

Supplement: Supplementary file 5 — 10.1186/s13072-015-0044-2 Global reactivation of the paternal X precedes the switching X-inactivation profiles in female TS cells. A. Cumulative histograms showing the expression levels of paternal (blue) and maternal (red) alleles of the indicated gene, assessed by allelic RT-qPCR, in parental F3 cells and in 3 independent clones showing low levels of Xist RNA. Two time points have been analysed: clones at passage 4 after clone picking and still under HAT pressure (Pass.4 histograms) and the same clones, 8 passages after HAT release (Pass.14 histograms). Note that, in contrast with other X-linked genes, Pbdc1 maintains a bi-allelic expression after HAT release in agreement with its status of XCI escaper. Standardisation by Rplp0 followed by standardisation by the median value for each gene have been applied to allow direct comparison of the different genes on the same histogram. For F3 parental female cells, mean and standard deviation have been calculated on the basis of three independent cell cultures. Above the histograms, the diagram depicts the scheme of the HAT selection experiment used to isolate HAT resistant clones analysed in this panel. A.U. : Arbitrary Units. B. Representative image of Xist RNA-FISH on nuclei of clone 2#C and clone 3#C maintained under HAT pressure. On the right, the histogram shows the percentages of nuclei of each depicted category. Clones under HAT pressure harbour significantly different Xist expression profiles compared to F3 parental cells (χ2 test p-value < 0.05). n > 150. [file 13072_2015_44_MOESM5_ESM.pdf]

**A**

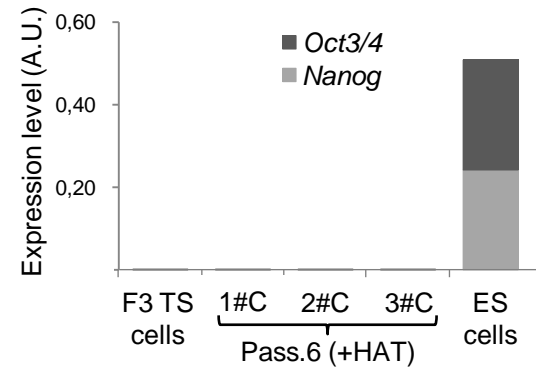

**B**

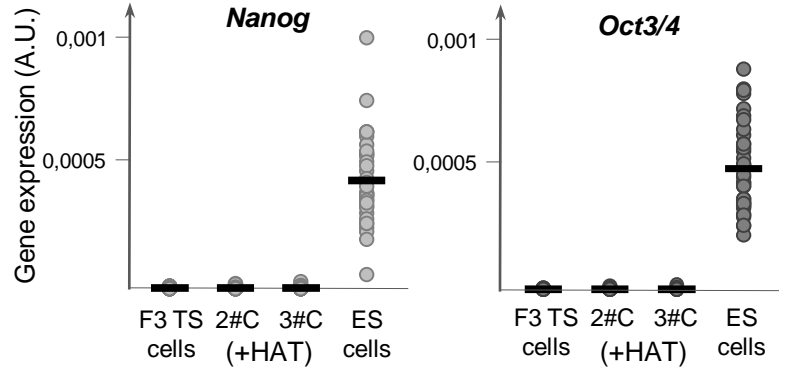

**C**

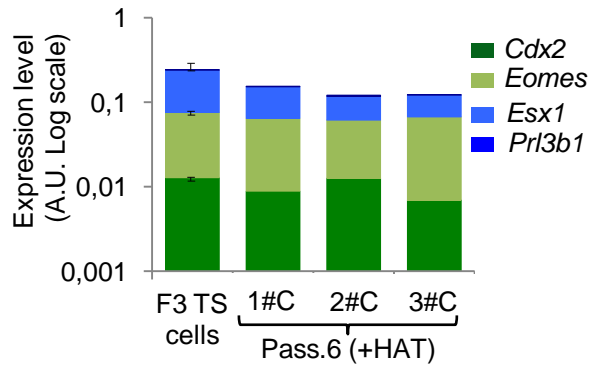

**D**

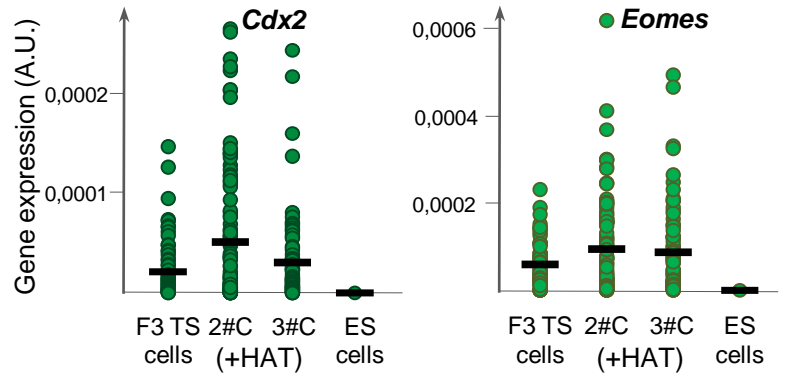

Supplement: Supplementary file 6 — 10.1186/s13072-015-0044-2 The two-active-X state of early HAT resistant clones is not associated with cell reprogramming towards pluripotency. A. Cumulative histograms showing the expression levels of pluripotency markers Nanog and Oct3/4 measured by RT-qPCR in F3 parental female TS cells and in 3 clones harbouring two active X-chromosomes. Expression levels in XX ES cells (LF2 cell line) are shown for comparison. Standardisation by Rplp0 has been applied. A.U.: Arbitrary Units. B. Same as in panel A on individual cells of each population. Small horizontal bars represent median values. See Additional file 7 for raw quantifications. C. Cumulative histograms showing the level of expression of TS specific markers Cdx2 and Eomes and of markers of TS differentiated states, Esx1 and Prl3b1 [19], assessed by RT-qPCR on a the same clones as in panel A. Expression levels have been standardised by Rplp0. Expression levels measured in F3 TS cells and in F3 TS cells differentiated for 5 days are shown for comparison. A.U. Arbitrary Units. Log scale is used. D. Same as in panel C for Cdx2 and Eomes on individual cells of each population. Small horizontal bars represent median values. See Additional file 7 for raw quantifications. [file 13072_2015_44_MOESM6_ESM.pdf]

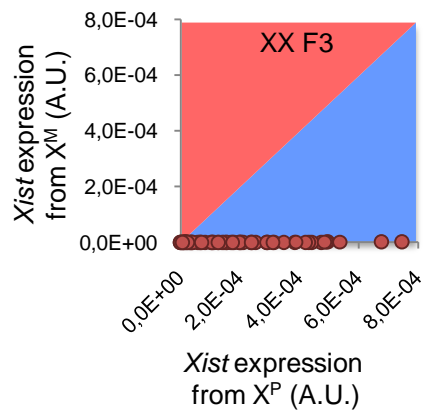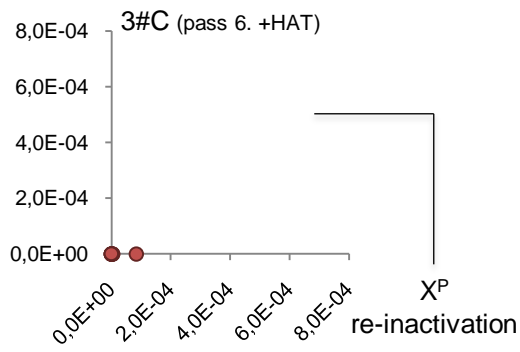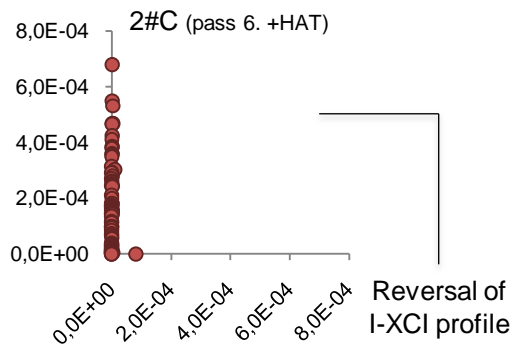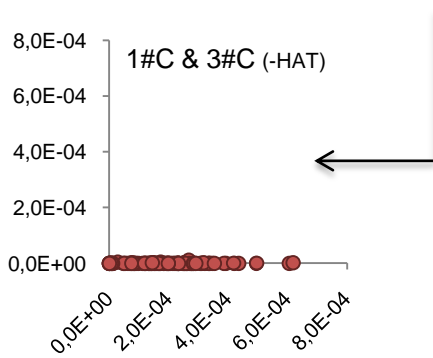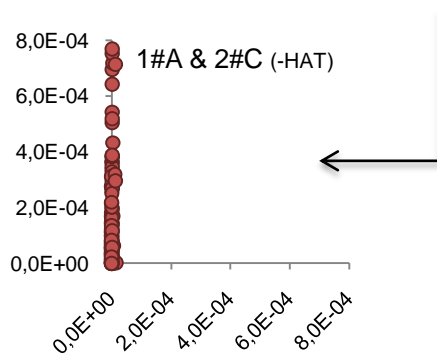

Supplement: Supplementary file 8 — 10.1186/s13072-015-0044-2 Single-cell analysis of Xist expression in clonal cell populations under HAT selection and after HAT removal. Scatter-plots of Xist expression levels from the paternal (x-axis) relative to the maternal (y-axis) X chromosome in individual cells of the indicated clones assessed by allelic RT-qPCR (BioMark, Fluidigm). Each dot represents a single TS cell. (+HAT): cells at passage 6 maintained under HAT pressure; (-HAT): cells after HAT removal. The same samples of cells have been analysed for the expression of the genes shown in Fig. 5 and in Additional file 9. For clones showing parental-like profiles or inverted I-XCI after HAT removal, cells from two different clones are shown. No significant difference was detected between cells of these two clones (Kolmogorov–Smirnov test). In clone 3#C under HAT pressure, one cells out of 74 tested show a significant Xist expression (> 0.5 10−5 A.U. corresponding to level of Xist RNA molecules in a Xist domain), while, in clone 2#C under HAT pressure, 55 cells out of 108 tested show a significant Xist expression. F3: n = 77; 3#C (+HAT): n = 75; 2#C (+HAT): n = 108; 1#C & 3#C (-HAT): n = 144; 1#A & 2#C (-HAT): n = 141. An additional table shows the raw quantification results (see Additional file 7). A.U.: Arbitrary Units. [file 13072_2015_44_MOESM8_ESM.pdf]

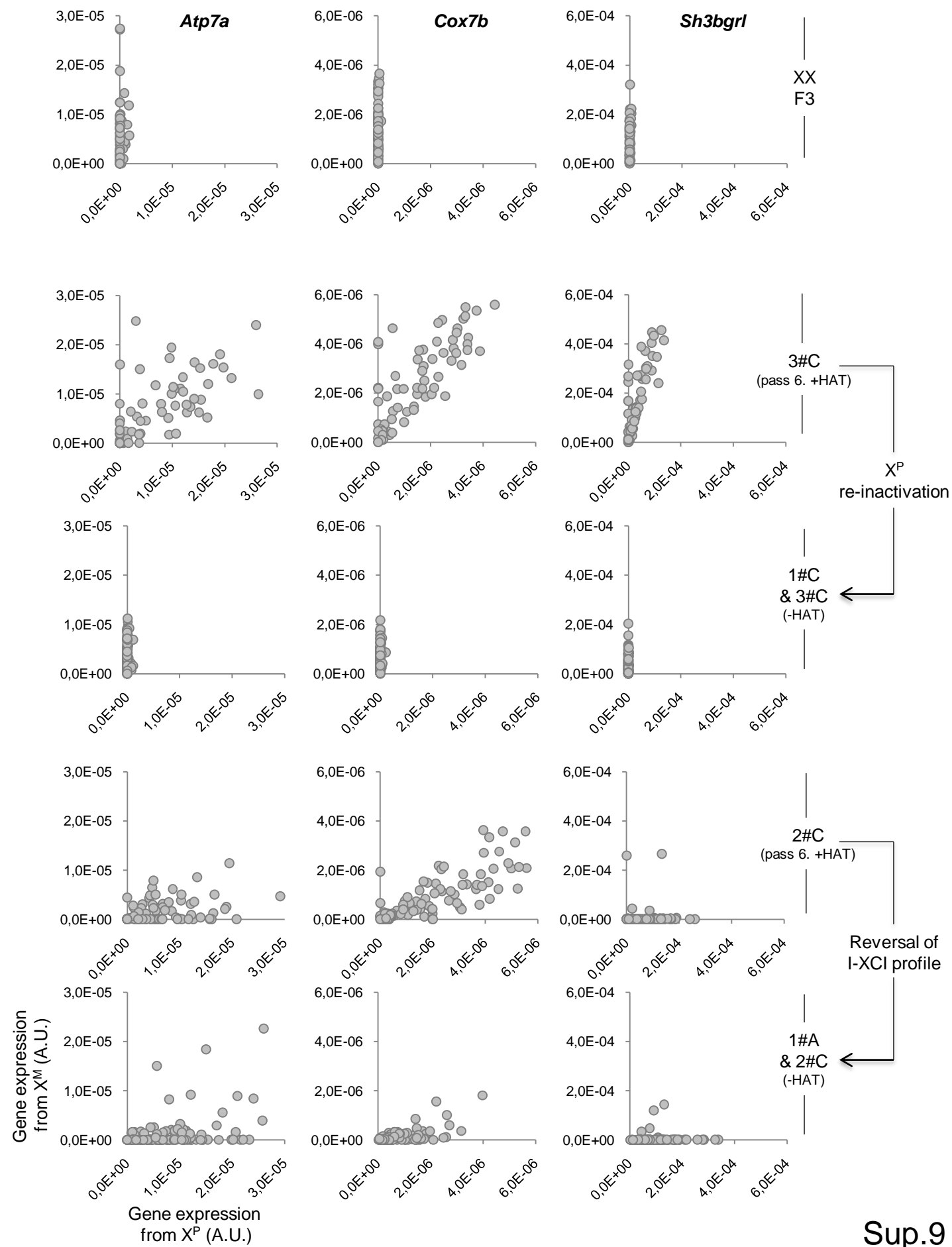

Supplement: Supplementary file 9 — 10.1186/s13072-015-0044-2 Single-cell analysis of gene expression of X-linked gene Atp7a, Cox7b and Sh3bgrl in clonal cell populations under HAT selection and after HAT removal. For details (see Additional file 8). See Additional file7 for raw quantifications. [file 13072_2015_44_MOESM9_ESM.pdf]
