# Supplementary material for: A rapid passage through a two-active-X-chromosome state accompanies the switch of imprinted X-inactivation patterns in mouse trophoblast stem cells
Source: Epigenetics Chromatin. 2015 Dec 1;8:52. doi: 10.1186/s13072-015-0044-2 (PMC4665903; doi:10.1186/s13072-015-0044-2)

A

	TS cell characteristics			Estimated frequency
	Xist expression	H3K27me3 accumulation	X-linked gene silencing	
F3 parental cells X^MX^P	domain on X^P in ~80% of cells X^MX^P	accum. on X^P in ~80% of cells X^MX^P	•Silencing of paternal alleles in most cells • Local reactivation of X^P-linked genes (incl. *Hprt1*) →	N/A ~0,7 10^{-5}
<u>HAT resistant clones cat.1</u> (+HAT) X^MX^P	domain on X^P in <10% of cells X^MX^P	ND	Bi-allelic at tested genes	~1,06 10^{-5}
"X^P re-inactivation" (-HAT) X^MX^P	domain on X^P in ~80% of cells X^MX^P	accum. on X^P in ~80% of cells X^MX^P	•Silencing of most paternal alleles • Local reactivation of X^P-linked genes (incl. *Hprt1*)	
<u>HAT resistant clones cat.2</u> (+HAT) X^MX^P	domain on X^M in ~50% of cells X^MX^P X^MX^P	ND	•Bi-allelic at *Cox7b*, *Atp7a* and *Pgk1* in ~50% of cells • Silencing of maternal *Sh3bgrl* and *Apool*	~8,62 10^{-7}
"X^M inactivation" I-XCI reversal (-HAT) X^MX^P	domain on X^M in >80% of cells X^MX^P	•accum. on X^M in >80% of cells X^MX^P • ~12% of genes escape H3K27me3	•Silencing of most maternal alleles in >80% of cells •Bi-allelic at H3K27me3-low genes	

B

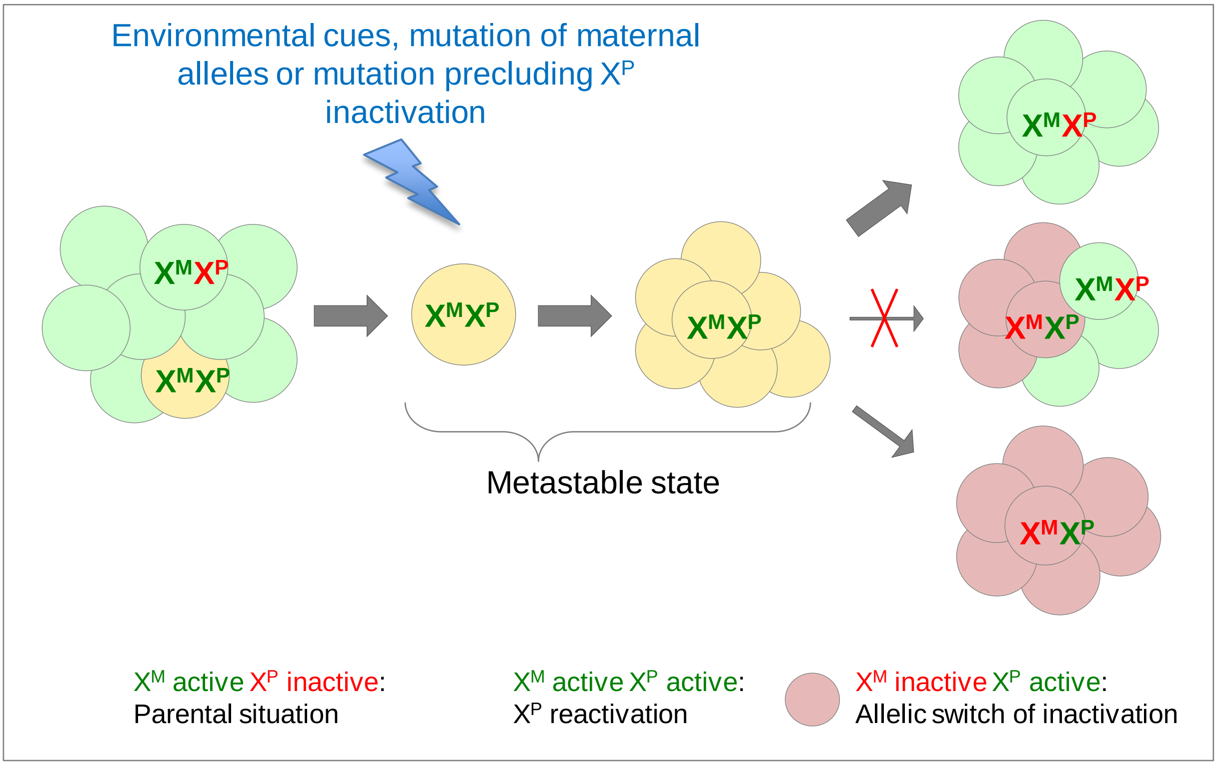

Supplement: Supplementary file 11 — 10.1186/s13072-015-0044-2 Summary of the metastable states of I-XCI in female TS cells observed in the present study. A. Table summarising the characteristics of the various TS cell populations observed in the study. B. We observe in the present study that undifferentiated TS cells spontaneously reactivate the paternal X partially or completely at a low frequency. Under specific environmental cues (including, in the case of the present study, the HAT selection) or mutation that either prevent XP inactivation or favour the expression of XP genes, TS cells carrying two active X chromosomes may undergo de novo inactivation of the maternal X. [file 13072_2015_44_MOESM11_ESM.pdf]
